# Tetra- and Penta-Acylated Lipid A Structures of *Porphyromonas gingivalis* LPS Differentially Activate TLR4-Mediated NF-κB Signal Transduction Cascade and Immuno-Inflammatory Response in Human Gingival Fibroblasts

**DOI:** 10.1371/journal.pone.0058496

**Published:** 2013-03-12

**Authors:** Thanuja D. K. Herath, Richard P. Darveau, Chaminda J. Seneviratne, Cun-Yu Wang, Yu Wang, Lijian Jin

**Affiliations:** 1 Faculty of Dentistry, Li Ka Shing Faculty of Medicine, The University of Hong Kong, Hong Kong SAR, China; 2 School of Dentistry, University of Washington, Seattle, Washington, United States of America; 3 School of Dentistry, University of California Los Angeles, Los Angeles, California, United States of America; 4 Department of Pharmacology & Pharmacy, Li Ka Shing Faculty of Medicine, The University of Hong Kong, Hong Kong SAR, China; University of California Merced, United States of America

## Abstract

**Background:**

*Porphyromonas gingivalis* is a major pathogen of periodontal disease that affects a majority of adults worldwide. Increasing evidence shows that periodontal disease is linked to various systemic diseases like diabetes and cardiovascular disease, by contributing to increased systemic levels of inflammation. Lipopolysaccharides (LPS), as a key virulent attribute of *P. gingivalis*, possesses significant amount of lipid A heterogeneity containing tetra- (LPS_1435/1449_) and penta-acylated (LPS_1690_) structures. Hitherto, the exact molecular mechanism of *P. gingivalis* LPS involved in periodontal pathogenesis remains unclear, due to limited understanding of the specific receptors and signaling pathways involved in LPS-host cell interactions.

**Methodology/Principal Findings:**

This study systematically investigated the effects of *P. gingivalis* LPS_1435/1449_ and LPS_1690_ on the expression of TLR2 and TLR4 signal transduction and the activation of pro-inflammatory cytokines IL-6 and IL-8 in human gingival fibroblasts (HGFs). We found that LPS_1435/1449_ and LPS_1690_ differentially modulated TLR2 and TLR4 expression. NF-κB pathway was significantly activated by LPS_1690_ but not by LPS_1435/1449_. In addition, LPS_1690_ induced significant expression of NF-κB and p38 MPAK pathways-related genes, such as NFKBIA, NFKB1, IKBKB, MAP2K4 and MAPK8. Notably, the pro-inflammatory genes including GM-CSF, CXCL10, G-CSF, IL-6, IL-8 and CCL2 were significantly upregulated by LPS_1690_ while down-regulated by LPS_1435/1449_. Blocking assays confirmed that TLR4-mediated NF-κB signaling was vital in LPS_1690_-induced expression of IL-6 and IL-8 in HGFs.

**Conclusions/Significance:**

The present study suggests that the tetra- and penta-acylated lipid A structures of *P. gingivalis* LPS differentially activate TLR4-mediated NF-κB signaling pathway, and significantly modulate the expression of IL-6 and IL-8 in HGFs. The ability to alter the lipid A structure of LPS could be one of the strategies carried-out by *P. gingivalis* to evade innate host defense in gingival tissues, thereby contributing to periodontal pathogenesis.

## Introduction

Periodontal disease is among the most common chronic infections and inflammatory events in humans, and severe periodontal disease (periodontitis) is the major cause of tooth loss in adults globally [1]. *Porphyromonas gingivalis* is considered a keystone bacterial pathogen strongly implicated in periodontal disease [2]–[4]. It is able to gain access to gingival tissues from pathogenic plaque biofilm and proliferate in gingival tissue, resulting in overt and unco-ordinated immuno-inflammatory response, and thereby leading to destruction of tooth supporting tissues [5], [6]. Lipopolysaccharide (LPS) is a cell wall component of Gram-negative bacteria including, *P. gingivalis*. This biomolecule is considered to be a major nexus for virulence in periodontitis [3], [7]. LPS basically consists of three segments with highly variable and conserved regions [8], [9]. They are a phosphorylated glucosamine disaccharide substituted with fatty acids known as lipid A which forms the matrix of the outermost membrane leaflet, a highly variable O-polysaccharide (O-antigen) and a conserved core oligosaccharide that links lipid A to the O-polysaccharide Lipid A section is the ‘bioactive centre’ of LPS, responsible for its endotoxicity. This is due to the specific and highly sensitive recognition of lipid A by host cells, which subsequently leads to strong immuno-inflammatory response [7], [9], [10].

*P. gingivalis* releases copious amounts of LPS that penetrates gingival tissues [11], [12] and actively participates in the pathogenic process of periodontitis [12]–[14]. Numerous studies in the past have examined the role of *P. gingivalis* LPS in periodontal pathogenesis. However, the precise nature of this relationship has been obscured due to lack of understanding of the underlying molecular mechanism of *P. gingivalis* LPS-host interaction. Some studies show that *P. gingivalis* LPS is a potent immune activator similar to the canonical *E. coli* LPS, whilst others report it to be immunologically inert [14], [15]. Hence, according to some studies *P. gingivalis* LPS induces pro-inflammatory cytokines [16], [17] whereas others argue that it may dampen the cytokine expression [18], [19].

Cell surface receptors and signal transduction pathways involved in *P. gingivalis* LPS and host cell interaction is at the heart of this long-standing debate. Most early studies with canonical *E. coli* LPS, containing hexa-acylated lipid A structure, have shown that *E. coli* LPS exclusively binds to toll-like receptor-4 (TLR4) [20], [21]. Although some claim that *E. coli* LPS may bind to TLR2, later studies showed that this was a result of lipoprotein contamination in LPS, since TLR2 is known to occupy the LPS ligand [22]. This controversy is further fuelled by the findings on LPS containing heterogeneous lipid A structures of non-enterobacterial species such as, *P. gingivalis, Bactereiodes fragilis* and *Pseudomonas aeruginosa* [23]–[26]. The common structural variation occurring in *P. gingivalis* LPS lipid A is due to the alteration of number of fatty acid chains attached to core disaccharide, which results in tetra- and penta-acylated structures [27], [28]. Hence, *P. gingivalis* LPS possesses lipid A structure containing both tetra-acylated (PgLPS_1435/1449_) and penta-acylated forms (PgLPS_1690_) compared to the hexa-acylated lipid A of *E. coli* LPS. Cell surface receptors and signal transduction pathways involved in host responses to aforementioned heterogeneous lipid A structures are the focus of the present study.

The heterogeneous nature of LPS lipid A renders *P. gingivalis* an unusual ability to interact with both TLR2 and TLR4, in contrast to *E. coli* LPS. Structural variation in lipid A moiety of *P. gingivalis* LPS may also differentially activate signal transduction pathways to elicit various immuno-inflammatory responses. For instance, hexa-acylated *E. coli* LPS preferentially activates TLR4-NF-κB cascade, whereas heterogeneous *P. gingivalis* LPS may use different cellular signaling pathways to modulate downstream pro-inflammatory cytokines [17], [29].

Controversial observations have been reported on *P. gingivalis* LPS-induced host response in various cell types that were investigated [30]. Most of the previous studies on *P. gingivalis* LPS have been performed in non-oral cells such as embryonic kidney cells, umbilical cord vein endothelial cells and monocytes [28], [29], [31], [32]. Only a few studies have undertaken on the primary cells of dental origin, which are more likely to interact with *P. gingivalis* LPS in clinical situations [33], [34]. Human gingival fibroblasts (HGFs) as the predominant structural cells in human gingiva represent a viable model to study *P. gingivalis* LPS-host interactions Firstly, HGFs express a number of pattern recognition receptors known to orchestrate immuno-inflammatory response [35]–[37]. Secondly, different isoforms of *P. gingivalis* LPS differently activate the expression of pro-inflammatory cytokines in HGFs as shown in our recent study [32]. Thirdly, HGFs play a pivotal role in the immuno-inflammatory response in the pathogenesis of periodontal disease [15], [38], [39].

The present study comprehensively investigated the effects of lipid A molecular heterogeneity of *P. gingivalis* LPS on the expression of TLR 2 and TLR4, downstream signal transduction and on the activation of pro-inflammatory cytokines in HGFs. *P. gingivalis* LPS_1435/1449_ and LPS_1690_ differentially modulated TLR2 and TLR4 expression. LPS_1690_ induced significant expression of NF-κB and p38 MPAK pathways-related genes as well as multiple pro-inflammatory genes. TLR4 and NF-κB were significantly involved in *P. gingivalis* LPS_1690_-induced expression of IL-6 and IL-8. Our findings demonstrate that *P. gingivalis* LPS with tetra- and penta-acylated lipid A structures differentially activate TLR4-mediated NF-κB signaling pathway, and critically modulate immuno-inflammatory response in HGFs.

## Results

### *P. gingivalis* LPS_1690_ and LPS_1435/1449_ Differentially Modulated the Expression of TLR2, TLR4 and MD2 Transcripts in HGFs

**Figure 1:**
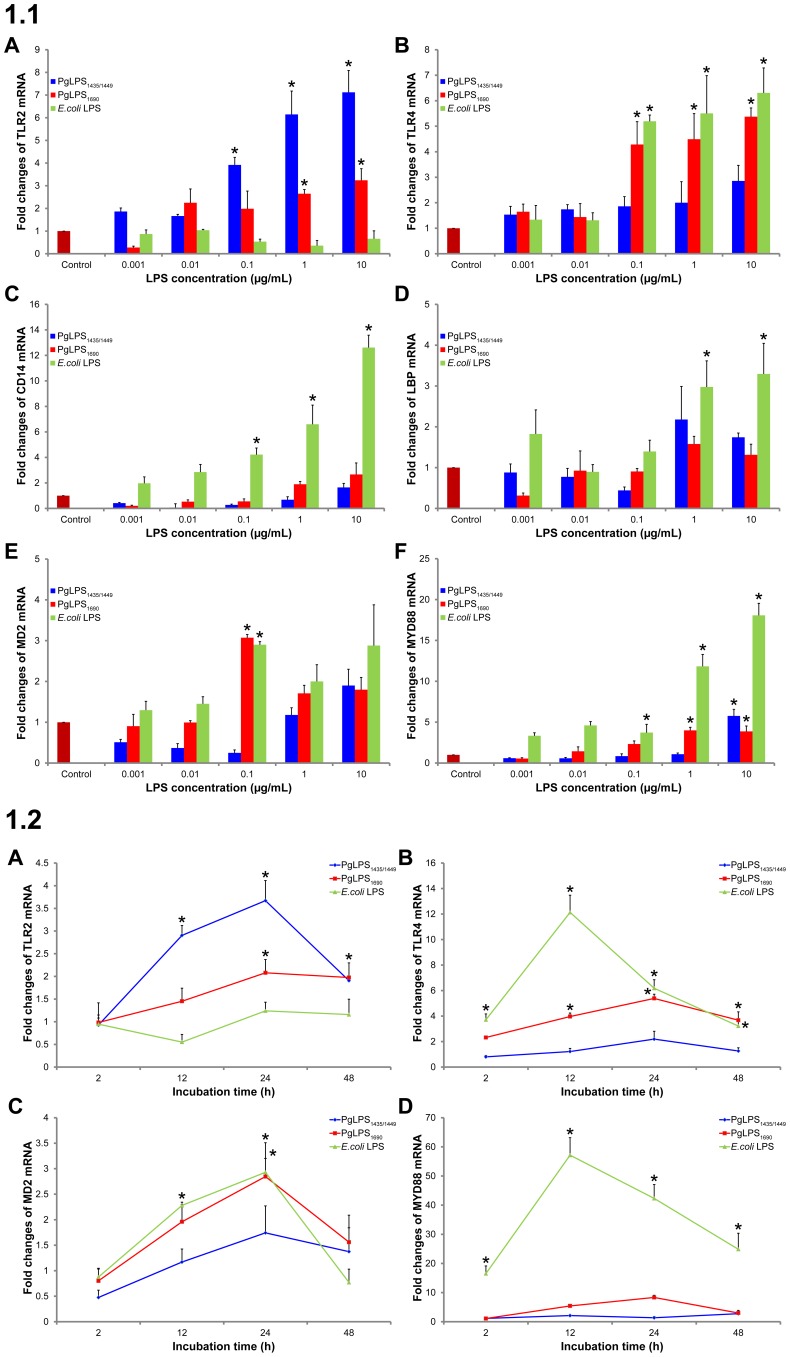
*P. gingivalis* LPS modulated the transcript expression of cell surface receptors and related co-molecules in HGFs. **1.1.** *P. gingivalis* (Pg) LPS_1690_ (PgLPS_1690_) and LPS_1435/1449_ (PgLPS_1435/1449_) differentially modulated the mRNA expression of TLR2 (A), TLR4 (B), CD14 (C), LBP (D), MD2 (E) and MYD88 (F) mRNAs in the cellular fractions of HGFs in the dose-dependent assay (1 ng/ml to 10 µg/ml) for 24 h. *E. coli* LPS is used as a reference. **1.2.** *P. gingivalis* LPS and *E. coli* LPS upregulated the expression of TLR2 (A), TLR4 (B), MD2 (C) and MYD88 (D) transcripts in the cellular fractions of HGFs. HGFs were treated with *P. gingivalis* (Pg) LPS (PgLPS) and *E. coli* LPS at 1 µg/ml in the time-dependent assay for 2 to 48 h. After LPS stimulation, the harvested RNAs were subjected to quantitative real-time PCR, and the fold changes in gene expression relative to internal control β-Actin were quantified as shown in the graphs. The mRNA expression of control was considered as 1. Each bar represents the mean ±SD of three independent experiments with three replicates. *Significant difference with a *p*-value <0.05 as compared with the controls without LPS treatment.

**Figure 2:**
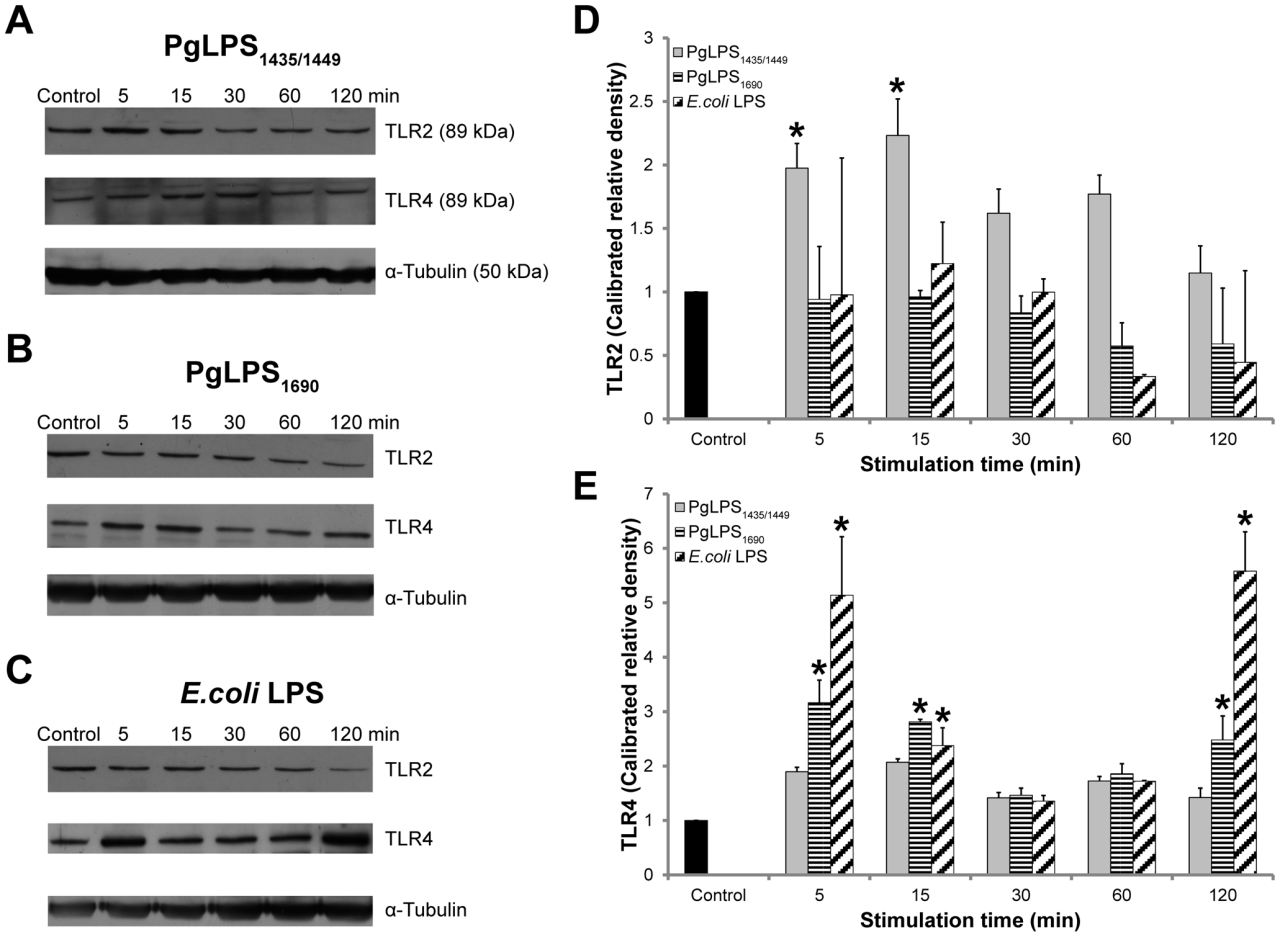
TLR2 and TLR4 protein expression in *P. gingivalis* LPS- and *E. coli* LPS-stimulated HGFs. Confluent HGFs were stimulated with *P. gingivalis* (Pg) LPS_1435/1449_ **(**PgLPS_1435/1449_) _1435/1449_ (A, D and E), PgLPS_1690_ (B, D and E) and *E. coli* LPS (C, D and E) (1 µg/mL) at the indicated time points in the western blot analysis for assay of TLR2 and TLR4 protein expression. 40 µg of homogenized cellular extracts were subjected to SDS-PAGE and probed with anti TLR2 (1∶1000) and anti-TLR4 (1∶1000) polyclonal antibodies. Blots were re-probed with tubulin to confirm equal loading in individual samples. One representative blot was shown from three independent experiments with similar results, TLR2∶89 kDa; TLR4∶96 kDa; and Tubulin: 50 kDa. Quantification of band intensities was performed by densitometry analysis using Image J software.

HGFs were treated with *E. coli* LPS and *P. gingivalis* LPS (LPS_1690_ and LPS_1435/1449_) in both dose- and time-dependent experiments to examine the transcript expression of TLR2, TLR4, MD2 and MyD88. Basal expression of both TLR2 and TLR4 could be observed in the untreated cells which was upregulated by *E. coli* LPS and *P. gingivalis* LPS (Figs. 1 and 2). *E. coli* LPS and *P. gingivalis* LPS_1690_ (not LPS_1435/1449_) significantly upregulated TLR4 expression at 0.1 µg/ml or above (Fig. 1.1B), and the expression level reached the peak at 12 and 24 h, respectively (Fig. 1.2B). Whereas, *P. gingivalis* LPS_1435/1449_ and to a less extent *P. gingivalis* LPS_1690_ significantly enhanced the TLR2 expression (Fig. 1.1A), and the peak expression was observed at 24 h (Fig. 1.2A). *E. coli* LPS significantly upregulated CD14 and LBP expression (Figs. 1.1C and D). MD2 was significantly upregulated by both *P. gingivalis* LPS_1690_ (not LPS_1435/1449_) and *E. coli* LPS (Figs. 1.1E and 1.2C). Additionally, MyD88 increased markedly by the stimulation of *E. coli* LPS and to a much less extent by *P. gingivalis* LPS (Figs. 1.1F and 1.2D). Foregoing data demonstrated that *P. gingivalis* LPS_1690_ and LPS_1435/1449_ could differentially modulate to a different extent the transcript expression of TLR2, TLR4 and MD2.

### *P. gingivalis* LPS_1690_ and LPS_1435/1449_ Differentially Modulated the Expression of TLR2 and TLR4 Proteins in HGFs

Next, in a time-course experiment (5–120 min) the expression of TLR2 and TLR4 proteins in HGFs was analyzed by western blot. Both TLR2 and TLR4 proteins were detected in all samples confirming their basal expression (Fig. 2). *P. gingivalis* LPS_1435/14495_ induced the prompt expression of TLR2 protein at 5 and 15 min (Figs. 2A and D). While there was a cyclic TLR4 expression pattern in cells treated with *P. gingivalis* LPS_1690_ and *E. coli* LPS, which was observed at 5, 15 and 120 min, respectively (Figs. 2B, C and E). These data further demonstrated that the expression of TLR2 and TLR4 in HGFs was differentially modulated by heterogeneous lipid A structures of *P. gingivalis* LPS. The expression profiles of TLR2 and TLR4 were further examined by antibody-mediated confocal immuno-fluorescence microscopy. HGFs showed basal expression of both TLR2 and TLR4. Whereas, *P. gingivalis* LPS_1435/1449 -_upregulated the basal expression of TLR2 at 6 and 24 h (Figs. 3.1 and S1.1). *P. gingivalis* LPS_1690_-upregulated expression of TLR2 was meager at 6 h (Fig. 3.1) and became more prominent at 24 h, and only a scanty expression was noted in cells treated with *E. coli* LPS at 24 h (Fig. S1.1). Relatively prompt and marked expression of TLR4 was observed in cells treated with PgLPS_1690_ and *E. coli* LPS at 6 h (Fig. 3.2) and to a less extent at 24 h (Fig. S1.2). These findings were overall consistent with foregoing results (Figs. 1 and 2). No positive signal was detected in negative controls, suggesting that the antibodies employed were actually bound to TLR2 and TLR4, and the non-specific binding or background staining was negligible.

**Figure 3:**
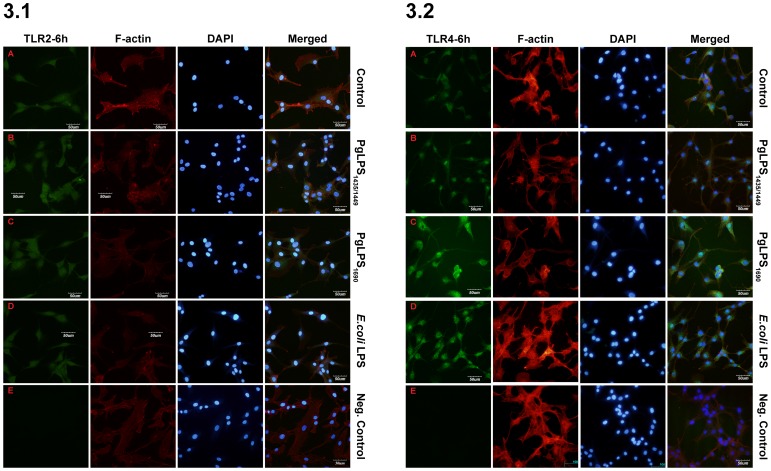
Confocal images showing positive TLR2 (3.1) and TLR4 (3.2) expression, in HGFs, following LPS stimulation. The cells were left untreated (A) or stimulated with *P. gingivalis* (Pg) LPS_1435/1449_ (PgLPS_1435/1449_) (B) PgLPS_1690_ (C) and *E. coli* LPS (D) at 1 µg/ml for 6 h. Cells were permeabilized with 0.1% Triton X-100 and subsequently stained with primary antibodies against TLR2, TLR4 and the correspondent secondary antibody labeled Alexa fluor 488 anti-rabbit, and subsequently stained with alexa fluor 555 phalloidin for F-actin. Merged images present the combined TLR2/TLR4, F-actin and nuclear staining (DAPI). Negative control: E. One representative experiment from three independent experiments is shown. Bar = 50 µm.

### The Expression Profiles of Genes Associated with TLR Signal Transduction in HGFs induced by *P. gingivalis* LPS_1690_ and LPS_1435/1449_

The potential modulation of other molecules involved in *P. gingivalis* LPS-induced TLR signaling pathway was analyzed using PCR gene-array. Both *P. gingivalis* LPS_1690_ and LPS_1435/1449_ significantly upregulated (fold changes ≥2.0) ELK1, HRAS, IL1B, TLR4, TLR5, TLR9, TNF, TRAF6 and UBE2N, and down regulated (fold changes <0.5) BTK, IL-2, IRAK1, LTA, CD180, MAPK8IP3, NFKBIL1, SIGIRR, TIRAP, TLR1 and TLR7 (Table S1). Notably, *P. gingivalis* LPS_1690_ markedly upregulated (≥3-folds) transcript levels of downstream pro-inflammatory genes, such as GM-CSF, CXCL10, G-CSF, IL-6, IL-8, CCL2 and TLR4, with reference to the untreated controls (Table 1 and Fig. S2). Moreover, *P. gingivalis* LPS_1690_ induced significant expression of NF-κB pathway-related genes such as NFKBIA, NFKB1 and IKBKB as well as p38 MPAK pathway molecules such as MAP2K4 and MAPK8 (Table 1). Interestingly, the following genes were differentially up- (fold changes from 2.26 to 26.77) or down-regulated (fold changes from 0.06 to 0.67) by the two isoforms of *P. gingivalis* (LPS_1690_ v.s. LPS_1435/1449_), respectively: GM-CSF (26.77 v.s. 0.28), CXCL10 (17.27 vs. 0.21), G-CSF (14.91 vs. 0.67), IL-6 (11.93 vs. 0.06), IL-8 (8.64 vs. 0.35), CCL2 (3.25 vs. 0.58) and CD14 (2.26 vs. 0.45). To confirm some of the strongly upregulated pro-inflammatory cytokine and chemokine genes (≥3-folds) by *P. gingivalis* LPS_1690_, the expression of GM-CSF, CXCL10, IL- 6 and IL- 8 transcripts were further validated by real-time qPCR (Fig. S3).

**Table 1:** List of genes upregulated (fold changes ≥1.5; highlighted in bold) and downregulated (fold changes ≤0.5; highlighted in italics) by *P. gingivalis* LPS_1435/1449_ and *P. gingivalis* LPS_1690_.

Genes	*P. gingivalis* LPS_1435/1449_	*P. gingivalis* LPS_1690_
CCL2	0.58	**3.25**
CXCL10	*0.21*	**17.27**
G-CSF	0.67	**14.91**
GM-CSF	*0.28*	**26.77**
IL6	*0.06*	**11.93**
IL8	*0.35*	**8.64**
HRAS	**4.66**	**6.74**
HSPA1A	1.28	**2.56**
TLR2	*0.24*	1.48
TLR4	**2.04**	**3.14**
CD14	*0.45*	**2.26**
IKBKB	**1.73**	**2.18**
NFKB1	1.48	**3.55**
NFKBIA	0.95	**4.25**
MAP2K4	1.38	**2.55**
MAP3K7IP1	**1.61**	**2.1**
MAP4K4	1.37	**1.71**
MAPK8	1.25	**1.99**
IRAK2	0.68	**2.25**

### *P. gingivalis* LPS_1690_ and LPS_1435/1449_ Differentially Determined the Activation of Intracellular Signal Transduction Pathways

**Figure 4:**
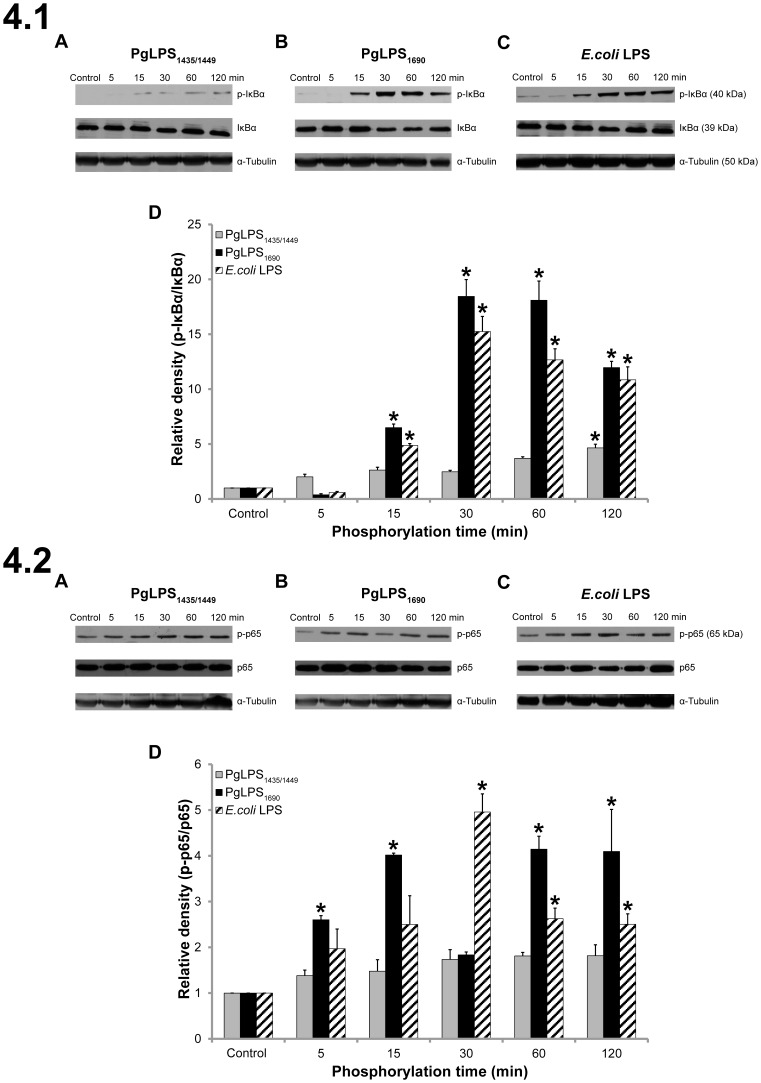
*P. gingivalis* (Pg) LPS_1690_ (PgLPS_1690_) and *E. coli* LPS activated the NF-κB pathway in HGFs. Kinetics of IκBα and NF-κB p65 phosphorylation in HGFs are shown in **4.1** and **4.2**, respectively. Cells were treated with PgLPS_1435/1449_ (A), PgLPS_1690_ (B) and *E. coli* LPS (C) at 1 µg/mL for the indicated period of time. Cell extracts were prepared and the levels of IκBα, phospho-IκBα, NF-κB p65, phospho-NF-κB p65 were determined by western blotting. Equal loading, for each treatment, was confirmed by stripping away the immunoblot, then re-probing it for α-Tubulin. Quantification of band intensities was performed by densitometry analysis using Image J software. The values for fold increase of phospho- IκBα (4.1D) and Phospho-NF-κB p65 (4.2D) as compared with the total protein are shown in the graphs (arbitrary units over control after normalization to the total protein). The data shown here are from a representative experiment repeated three times with similar results**.** *Significant difference with a *p*-value <0.05 as compared with the controls without LPS treatment.

The activation of NF-κB and MAPK signal pathways were examined by western blot in HGFs in response to the different isoforms of *P. gingivalis* LPS_1690_ and LPS_1435/1449_. As shown in Fig. 4, *P. gingivalis* LPS_1690_ and *E. coli* LPS induced the phosphorylation of IκBα and the p65 subunit of NF-κB. Both induced intense phosphorylation of IκBα after 15 min stimulation, which remained to be activated at 120 min (Figs. 4.1B–D). Comparably, *P. gingivalis* LPS_1435/1449_ induced only a weak activation of IκBα (Fig. 4.1A and D). There was a considerable level of constitutive expression of phosphorylated p65 in HGFs and the upregulation of p-p65 was marginal. However, activation of p65 subunit was observed promptly after 5 min stimulation of *P. gingivalis* LPS_1690_ and 30 min stimulation of *E. coli* LPS (Figs. 4.2B–D). No significant phosphorylation of p65 was activated by *P. gingivalis* LPS_1435/1449_ (Fig. 4.2A). Both *P. gingivalis* LPS_1690_ and LPS_1435/1449_ as well as *E. coli* LPS induced phosphorylation of p38 MAPK (Figs. 5.1A–C). *P. gingivalis* LPS_1690_ activated p38 MAPK at 15 min which lasted consistently until 120 min (Figs. 5.1B and D). Whereas, *P. gingivalis* LPS_1435/1449_ promptly activated the phosphoryaltion of p38 MAPK at 5 min and it remained significant until 120 min (Figs. 5.1A and D). Both *P. gingivalis* LPS and *E. coli* LPS activated ERK1/2 in a similar manner (Figs. 5.2). On the other hand, SAPK/JNK was not significantly activated by *P. gingivalis* LPS and *E. coli* LPS (Fig. 5.3). Similarly, there was no significant activation of AKT pathway upon stimulation with the two isoforms of *P. gingivalis* LPS (Figs. S4 A, B and D). In contrast, *E. coli* LPS significantly induced AKT phosphorylation at 30 min (Figs. S4 C and D). These data demonstrated that the structural heterogeneity of *P. gingivalis* LPS could determine the activation of signal transduction pathways in HGFs. Hence, penta-acylated *P. gingivalis* LPS_1690_ significantly activated NF-κB, p38 MAPK and ERK1/2 signals, but not the SAPK/JNK and AKT pathways. Similarly, the hexa-acylated *E. coli* LPS activated all aforementioned signaling pathways other than SAPK/JNK. In contrast, tetra-acylated *P. gingivalis* LPS_1435/1449_ predominately activated p38 MAPK and ERK1/2 signals, but did not strongly induce the NF-κB pathway.

**Figure 5:**
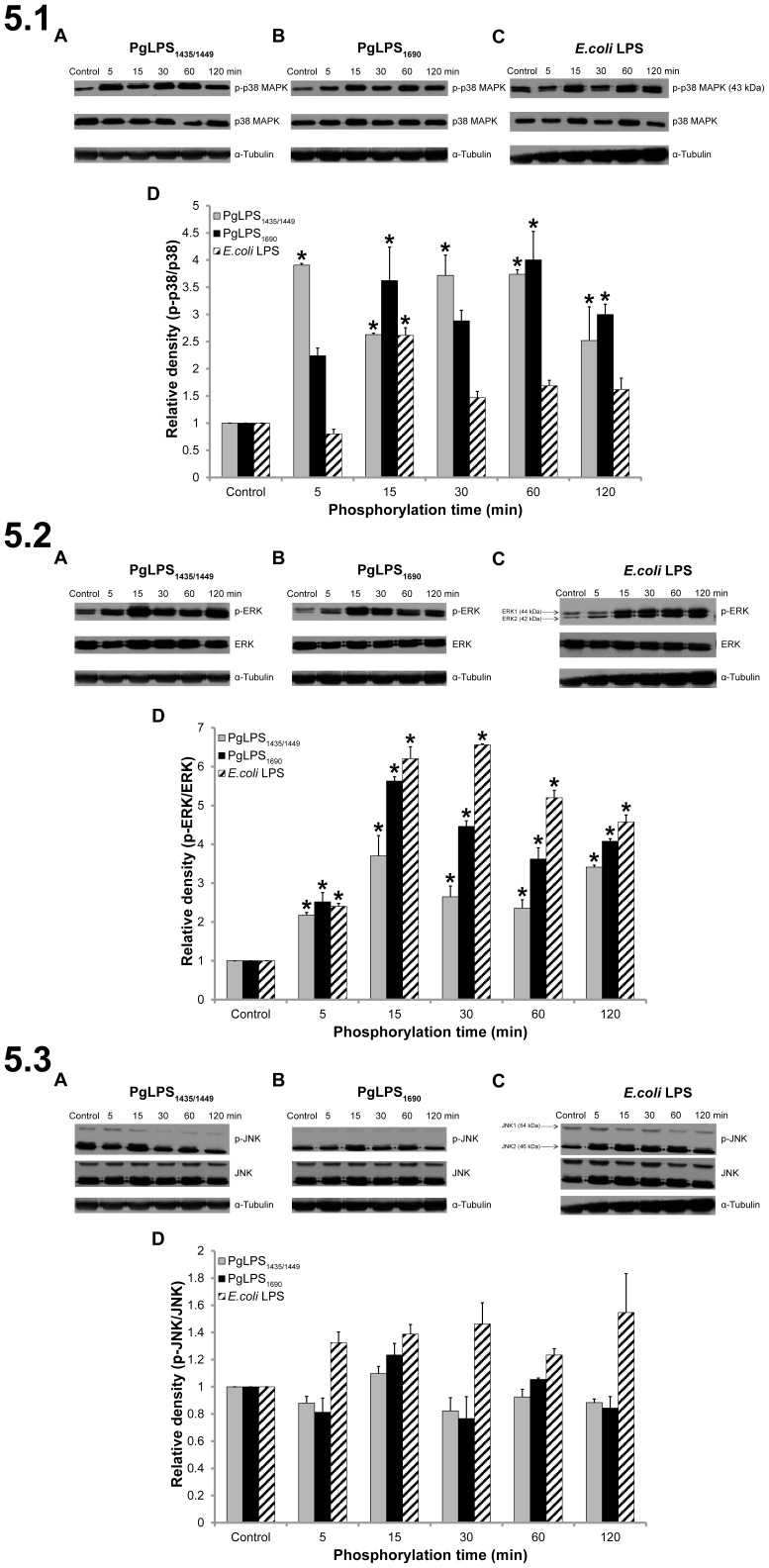
*P. gingivalis* (Pg) LPS (PgLPS) and *E. coli* LPS activated the MAPK pathway in HGFs. Kinetics of P38 mitogen activated protein kinase (P38 MAPK), extracellular signal-regulated kinase1/2(ERK1/2), and Stress-activated protein kinase/c-Jun NH2-terminal kinase (SAPK/JNK) phosphorylation in HGFs are shown in **5.1**, **5.2** and **5.3**, respectively. Cells were treated with PgLPS_1435/1449_ (A), PgLPS_1690_ (B) and *E. coli* LPS (C) at 1 µg/mL for the indicated period of time. Cell extracts were prepared and the levels of P38 MAPK, phospho-p38MAPK, ERK, phospho-ERK, JNK and phospho-JNK were determined by western blotting. Quantification of band intensities was performed by densitometry analysis using Image J software. The fold increase values of phospho-protiens of P38 MAPK (5.1D), ERK1/2 (5.2D) and SAPK/JNK (5.3D) as compared with the total protein are shown in the graphs (arbitrary units over control after normalization to the total protein). Equal loading was confirmed by stripping the immunoblot and re-probing it for α-Tubulin. The data shown here are from a representative experiment repeated three times with similar results**.** *Significant difference with a *p*-value <0.05 as compared with the controls without LPS treatment.

### *P. gingivalis* LPS_1690_ Induced p-p65-NF-κB Nuclear Translocation in HGFs

**Figure 6:**
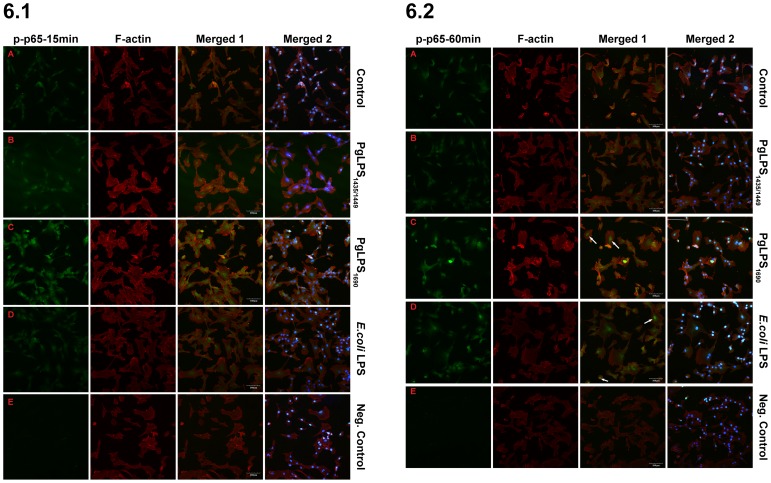
Confocal images of p-p65 NF-κB nuclear translocation in LPS treated HGFs. The cells were left untreated (A) or stimulated with *P. gingivalis* (Pg) LPS_1435/1449_ **(**PgLPS_1435/1449_) (B), PgLPS_1690_ (C) and *E. coli* LPS (D) (1 µg/ml) for 15 min (**6.1**) and 60 min (**6.2**), respectively. Cells were permeabilized with 0.1% Triton X-100 and subsequently stained with primary antibodies against anti-phospho p65-NF-κB and the correspondent secondary antibody labeled Alexa fluor 488 anti-rabbit, and subsequently stained with alexa fluor 555 phalloidin for F-Actin. The cytoplasmic p-p65 NF-κB appears in green color and F-actin is shown in red color. Negative control: E. The arrow heads show the prominent nuclear staining in the nucleus. Merged 1 images present the combined p-p65- NF-κB and F-actin, whereas Merged 2 images show the combined p-p65-NF-κB, F-actin and nuclear staining which is counterstained with DAPI. The experiment was performed three times, and the pictures observed correspond to a representative field for each of the times studied. Scale bar = 100 um.

Nuclear translocation of phospho-NF-κB p65 was observed using confocal immuno-fluorescence microscopy. p-p65-NF-κB translocation was prominent in *P. gingivalis* LPS_1690_-treated cells as compared with both untreated control and *P. gingivalis* LPS_1435/1449_ (Fig. 6). At the early stage (15 min), p65 was mainly present in the cytoplasm, and the subsequent translocation took place within 60 min following *P. gingivalis* LPS_1690_ stimulation (Figs. 6.1 and 6.2). In the normal condition, p65-NF-κB is retained in the cytosol in an inactive state being complexed with the inhibitory protein IκBα. However, upon stimulation with LPS, p-p65-NF-κB translocates to the nucleus following the gradual degradation of IκBα. Here we observed that p65-NF-κB was evenly distributed in the cytoplasm in untreated control cells without the sign of p65 immunoreactivity (Fig. 6.1A). However, nuclear expression of p-p65-NF-κB observed in controls after 60 min could be due to the increased intensity with longer exposure time rather than the translocation (Fig. 6.2A). Following the stimulation with *P. gingivalis* LPS_1690_ and *E. coli* LPS for 15 min, p-p65-NF-κB started to migrate to the perinuclear area and the translocation was completed within 60 min (Figs. 6.1C & D and 6.2C & D). These data suggested that *P. gingivalis* LPS_1690_ could induce the nuclear translocation of p-p65 which could be important for the optimal transcription of NF-κB dependent genes.

### Functional Involvement of TLR2 and TLR4 in *P. gingivalis* LPS_1690_-induced Expression of IL-6 and IL-8

Blocking assays were used to determine the functional involvement of TLR2 and TLR4 in *P. gingivalis* LPS – HGFs interactions by measuring the expression of downstream cytokines such as, IL-6 and IL-8. We previously demonstrated that *P. gingivalis* LPS_1690_ (not *P. gingivalis* LPS_1435/1449_) and *E. coli* LPS induced significant expression of IL-6 and IL-8 in HGFs [33]. Blockage of TLR4 significantly inhibited the *P. gingivalis* LPS_1690_- and *E. coli* LPS-induced expression of IL-6 and IL-8 mRNAs (Fig.7.1) and proteins (Fig. 7.2). Whereas, blockage of TLR2 led to significant inhibition of *P. gingivalis* LPS_1690_-induced expression of IL-6 mRNA and protein (Figs. 7.1A and 7.2A), as well as IL-8 mRNA (Fig. 7.1B). It could therefore be assumed that *P. gingivalis* LPS_1690_ may induce the expression of pro-inflammatory cytokines like IL-6 via both TLR2 and TLR4, which may be in a way different from *E. coli* LPS with its hexa-acylated lipid A structure.

**Figure 7:**
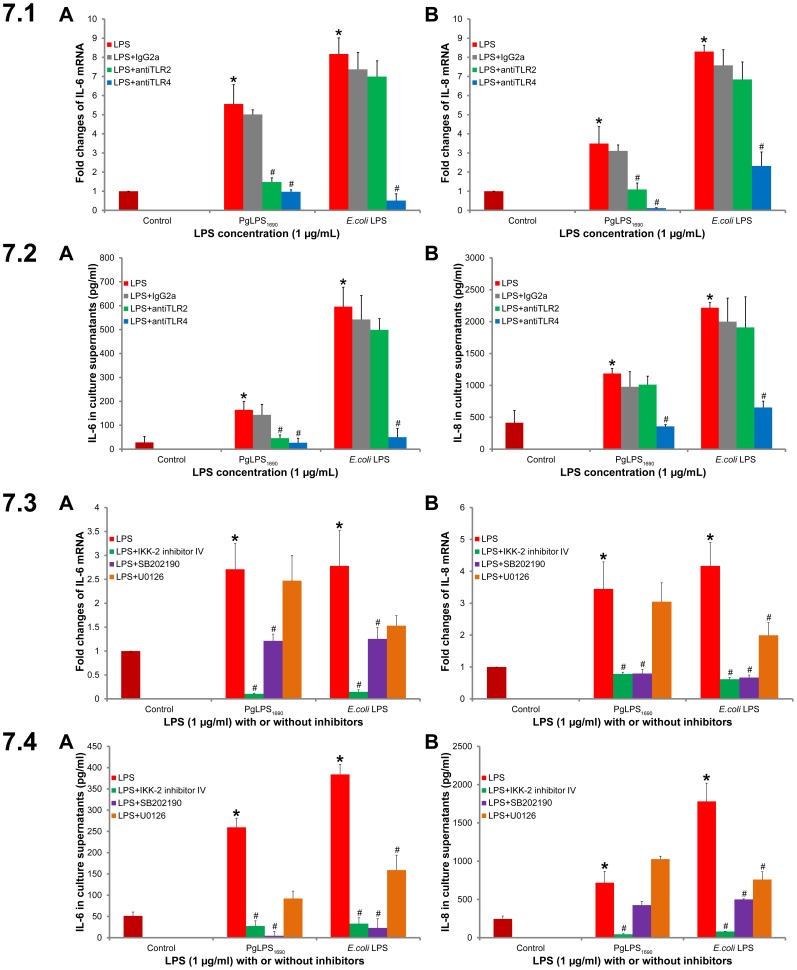
Blocking assay on the involvement of TLR2/TLR4 and signal transduction pathways in *P. gingivalis* (Pg) LPS_1690_ (PgLPS_1690_)- and *E. coli* LPS-induced expression of IL-6 and IL-8 in HGFs. The cells were pretreated for 1 h with anti-TLR2 and anti-TLR4 antibodies in serum free medium, and then treated with PgLPS and *E. coli* LPS at 1 µg/ml for additional 12 h. Total RNA and cell culture supernatants were collected and analyzed for IL-6 (A) and IL-8 (B) by quantitative real-time PCR and ELISA, respectively. The histograms show IL-6 (7.1A) and IL-8 (7.1B) mRNA levels of three independent experiments, and IL-6 (7.2A) and IL-8 (7.2B) protein expression levels of two independent experiments. The results were presented as mean ±SD. Calculation of significant difference were made in comparison to the controls without LPS treatment (*p-value <0.05) or the cells treated with LPS alone (#*p*-value <0.05). Cells were pretreated with IKK-2 inhibitor IV (IKK-β inhibitor), SB202190 (p38 MAPK inhibitor) and U0126 (ERK inhibitor) in serum free medium for 1 h, then treated with PgLPS and *E. coli* LPS at 1 µg/ml for additional 12 h. The histograms show IL-6 (7.3A) and IL-8 (7.3B) mRNA levels of three independent experiments, and IL-6 (7.4A) and IL-8 (7.4B) protein expression levels of two independent experiments. The results were presented as mean±SD. Calculation of significant difference were made in comparison to the controls without LPS treatment (*p-value <0.05) or the cells treated with LPS alone (#*p*-value <0.05).

### NF-κB Pathway Played a Crucial Role in *P. gingivalis* LPS_1690_-induced Expression of IL-6 and IL-8 in HGFs

Pathway-specific blocking assays further determined the involvement of signal transduction pathways in *P. gingivalis* LPS-induced IL-6 and IL-8 expression in HGFs. Specific kinase inhibitors were used, including IKK-β inhibitor (IKK-2 inhibitor IV), p38 MAPK (SB202190) and ERK kinase MEK-1 (U1026). The IKK inhibitor significantly attenuated the expression of IL-6 mRNA and protein (Figures 7.3A and 7.4A) as well as IL-8 mRNA and protein (Figs. 7.3B and 7.4B) induced by *P. gingivalis* LPS_1690_ and *E. coli* LPS. The p38 MAPK inhibitor blocked, to a different extent, *P. gingivalis* LPS_1690_- and *E. coli* LPS-stimulated expression of IL-6 and IL-8 (Figs. 7.3 and 7.4). ERK inhibitors did not significantly affect the expression of these cytokines induced by *P. gingivalis* LPS_1690_; while, ERK was significantly involved in *E. coli* LPS-induced expression of IL-6 protein (Fig. 7.4A) as well as IL-8 mRNA and protein (Figs. 7.3B and 7.4B). These data revealed that NF-κB and likely p38 MAPK signaling pathways may play a crucial role in *P. gingivalis* LPS_1690_ induction of pro-inflammatory cytokines, which was different from *E. coli* LPS where NF-κB, p38 MAPK and ERK transduction pathways were, to a different extent, significantly involved in induction of the cytokine expression.

## Discussion

It is evident that LPS as the prototypical endotoxin from gram-negative bacteria is highly potent in inducing innate host response [40]. Over the years, the crucial role of *P. gingivalis* LPS in the pathogenesis of periodontal disease has been intensively investigated [3], [4], [6], [10]–[19]. Whereas, the exact cell surface receptor for *P. gingivalis* LPS has long been a subject of intense debates, as some studies show the involvement of TLR4, whereas others argue it to be TLR2 [24], [41], [42]. Similar controversy exists over the major signal transduction pathways involved in immuno-inflammatory response to *P. gingivalis* LPS, as some suggest it to be NF-κB pathway whilst others propose the role of MAPK signal transduction [30]. Complicating this issue further, some studies indicate the involvement of both NF-κB and MAPK pathways as well as other additional signal pathways like JNK or AKT [14]. The discovery of lipid A heterogeneity of *P. gingivalis* LPS and the contrasting biological activities of its different isoforms, including LPS_1435/1449_ and LPS_1690_, shed new light on this confounding issue [28], [42], [43]. In an *in vivo* study in mice, the two isoforms stimulated local and systemic inflammatory responses in a different manner, presumably due to the complex nature of the local and systemic host responses [44]. Moreover, it has been observed that TLR2 could be an important determinant in response to *P. gingivalis in vivo* [45] and induce inflammatory destruction of bone in mice [46]. In addition, studies using *P. gingivalis* as a whole bacterium have shown CD14-TLR1-TLR2 complex is important to gain access to the cells [47]. Regarding the *in vitro* studies, there is a lack of consistently strong evidence on the cell surface receptors and signal transduction pathways that are involved in the interaction of heterogeneous *P. gingivalis* lipid A structures in host cells such as HGFs [14], [24], [30], [41], [42]. The present study attempted to examine the effects of *P. gingivalis* LPS_1435/1449_ and LPS_1690_ on the expression of TLR 2 and TLR4, downstream signal pathways involved and the expression of pro-inflammatory cytokines in HGFs.

Our present study revealed that LPS containing penta- and hexa-acylated lipid A structures, which were represented by *P. gingivalis* LPS_1690_ and *E. coli* LPS, upergulated strong expression of TLR4 in HGFs in both dose- and time-dependent manners, although the former also activated the expression of TLR2. On the other hand, tetra-acylated *P. gingivalis* LPS_1435/1449_ predominantly upregulated the expression of TLR2, and weakly increased the expression of TLR4. These observations were further confirmed by western blot analysis and confocal immuno-fluorescence microscopy. Blocking assays demonstrated that TLR4 was a critical receptor in immuno-inflammatory response to penta-acylated *P. gingivalis* LPS_1690_ and hexa-acylated *E. coli* LPS. Moreover both forms of LPS activated NF-κB, p38 MAPK and ERK pathways, but not the SAPK/JNK pathway. Additionally, *E. coli* LPS could activate AKT signal. On the contrary, *P. gingivalis* LPS_1435/1449_ activated to a different extent p38 MAPK and ERK1/2 signals. Taken together, these findings demonstrated that *P. gingivalis* LPS stimulated an overall different expression profile of TLR2 and TLR4 as well as the downstream signaling from that stimulated by the canonical *E. coli* LPS. It has been shown that five of the six fatty acid chains of *E. coli* LPS lipid A could occupy the pocket created by TLR4-MD2 complex that was crucial for TLR4 dimerization and activation of subsequent signaling pathways [40]. As *P. gingivalis* LPS_1690_ and LPS_1435/1449_ differentially stimulated the expression profiles of TLR2 and TLR4, the tetra-acylated lipid A structure of the latter might either fill the space available in the pocket or make varied changes to the complex by nullifying the effect of corresponding LPS ligand [40]. Further investigation is required to clarify this point.

Moreover, we also found that *P. gingivalis* LPS_1690_ induced the nuclear translocation of p-p65, which is critical in the optimal transcription of NF-κB-dependent genes such as IL-6 and IL-8 [48]. Further blocking assays confirmed that NF-κB pathway played a dominant role in induction of IL-6 and IL-8 in HGFs in response to *P. gingivalis* LPS_1690_ and *E. coli* LPS. These findings could be further discussed in the context of existing literature on the interaction between *P. gingivalis* LPS and host cells. Interaction of *P. gingivalis* LPS with human embryonic kidney cells involves both TLR2 and TLR4, whereas *Salmonella minnesota* LPS is only sensed by TLR4 [28]. Incidentally, later studies reveal that aforementioned *P. gingivalis* LPS could be a mixture of both tetra- and penta-acylated lipid A structures [31]. Hence, the biological activity of penta-acylated lipid A structure of *P. gingivalis* LPS seems to mimic that of canonical hexa-acylated lipid A structure of *E. coli* LPS. It has been demonstrated that penta-acylated lipid A molecules from various Gram-negative bacteria can interact with TLR4, compete and antagonize the action of hexa-acylated *E. coli* LPS [49]. A similar line of observations has been made with heterogeneous lipid A structures of *P. gingivalis* LPS that antagonize the inflammatory response by competing for TLR4 occupation in human umbilical vein endothelial cells (HUVECs) [50]. On the other hand, some studies have shown that TLR2 receptor could be involved in host cell recognition of *P. gingivalis* LPS [28], [51], [52]. The expression of IL-6 in cementblasts in response to *P. gingivalis* LPS_1690_ and *P. gingivalis* LPS_1435/1449_ is inhibited by blockage of TLR2, but not TLR4 [51]. There is a strong activation of NF-κB pathway in response to *P. gingivalis* LPS_1690_ with reference to a weak activation of *P. gingivalis* LPS_1435/1449_, illustrating the significant role of lipid A structure in activation of NF-κB pathway. The host response of dental pulp cells to *P. gingivalis* LPS is also elicited via TLR2/IKK signal transduction axis [53]. Although, it seems that *P. gingivalis* LPS, being different from canonical *E. coli* LPS, may have some propensity to bind TLR2, some have previously argued that it could be due to the contamination of lipoprotein or other components during LPS extraction. However, recent studies demonstrated that highly purified *P. gingivalis* LPS facilitates activation of both TLR2 and TLR4 in various host cell types [28]. In addition, extensively purified *P. gingivalis* LPS stimulates TLR2 expression [23], [54], [55]. A study has compared the functional effects of highly purified endotoxins from *E. coli*, *P. gingivalis*, *Pseudomonas aeruginosa* and *Bacteroides fragilis* in HUVECs and coronary artery endothelial cells (HCAECs). It shows that HCAEC’s which express TLR2 are responsive to LPS from species other than *E. coli*. It is therefore conceivable that *E. coli* LPS solely utilizes TLR4, whilst LPS from other bacterial species may utilize TLR2 as well [55]. Taking data from foregoing studies and the data derived from the present study into consideration, it shows that although both isoforms of *P. gingivalis* LPS could activate TLR2 expression, *P. gingivalis* LPS_1690_ is a strong activator of TLR4 expression, whereas *P. gingivalis* LPS_1435/1449_ is just a weak agonist for TLR4. The hexa-acylated *E. coli* LPS is then a potent agonist for TLR4.

Previous studies have reported that *P. gingivalis* LPS_1690_ could be a strong inducer for NF-κB pathway through TLR4 signaling in HEK293 cells and endothelial cells [56]. In contrast, *P. gingivalis* LPS_1435/1449_ does not elicit a significant immuno-inflammatory activity [56], [57]. We have recently demonstrated that *P. gingivalis* LPS_1690_ is an active inducer of pro-inflammatory cytokines in HGFs, whilst *P. gingivalis* LPS_1435/1449_ is unable to activate the response [33]. Findings of the present study may explain the mechanism behind this observation. Hence, *P. gingivalis* LPS_1435/1449_ that does not strongly activate TLR4 expression and NF-κB signals is less potent for immuno-stimulation as compared to the more potent isoform of penta-acylated *P. gingivalis* LPS_1690_,which significantly activates NF-κB pathway similar to that of *E. coli* LPS. This notion may explain the ability of hexa-acylated *E. coli* LPS and penta-acylated *P. gingivalis* LPS_1690_ to induce the NF-κB pathway and its downstream pro-inflammatory cytokines in a way different from the tetra-acylated *P. gingivalis* LPS_1435/1449_.

Our study shows that *E. coli* LPS and *P. gingivalis* LPS_1690_, to a different extent induced CD14 expression in HGFs. Although CD14 is known as a receptor for LPS binding, its precise role in *P. gingivalis* LPS-host interaction remains undefined [14], [28], [36], [58]. Some have reported that HGFs do not express membrane-bound CD14 whilst others show the reverse [59], [60]. Hence, CD14 may not critically involve in the interaction of HGFs with *P. gingivalis* LPS with reference to toll-like receptors as shown above. The observation that LBP mRNA is significantly upregulated in *E. coli* LPS treated HGFs as compared to cells treated with *P. gingivalis* LPS corroborates the previous finding that the binding capacity of *E. coli* LPS to LBP is much stronger than binding of *P. gingivalis* LPS [23].

Our current findings on structure-function relationship of LPS lipid A component have both biological and clinical implications. Conventionally, it is assumed that hexa-acylated lipid A from canonical *E. coli* LPS is bound to LBP, which is transferred to CD14 and then to TLR4/MD2 complex. This receptor binding subsequently triggers oligomerization and translocation of NF-κB into the nucleus, leading to secretion of pro-inflammatory cytokines [61]. However, structural variation of lipid A molecule could bring about different types of biological interaction of LPS with host cells. Previous studies have shown that modification of canonical *E. coli* lipid A structure, by replacing C12 fatty acid (laurate) with long-chain C16 (palmitate), results in less potent LPS [62]. Hence, length and number of fatty acid chain could significantly modulate the activation of host signal transduction and the resultant immuno-inflammatory response [43], [62], [63]. The present study demonstrates that structural heterogeneity in *P. gingivalis* LPS lipid A is a critical determinant of host cell sensing and signaling towards pathogens. The molecular conformation of lipid A structure has been shown to influence the supra-molecular structure of LPS, *i.e.* cylindrical lipid A generates lamellar structures whilst conical lipid A forms cubic or hexagonal structures [64], [65]. Therefore, three-dimensional arrangement of lipid A is a crucial determinant of LPS activity. As the number of attached fatty acid chains in the lipid A decreases, so does the potency of LPS. *E. coli* lipid A. So with a conical shape consisting of six asymmetrical acyl chains, *E. coli* lipid A is a potent activator of immuno-inflammatory response, while *P. gingivalis* LPS lipid A comprising of four-acyl chains such as, *P. gingivalis* LPS_1435/1449_ has strictly cylindrical conformations, which result in relatively weak activity to induce host response. Not only periodontal pathogens like *P. gingivalis*, but also other Gram-negative bacterial species such as *Rhodobacter capsulatus* or *Chromobacterium violaceum* contain tetra-acylated lipid A structures which are weak inducers of pro-inflammatory mediators like IL-6 [66].

Three-dimensional conformation due to variation in lipid A structure could elegantly explain the differential biological activity of *P. gingivalis* LPS lipid A component, in terms of receptor binding and subsequent activation of signal transduction cascades. For instance, conical shape *E. coli* LPS bearing hexa-acylated lipid A exclusively binds to TLR4, whereas less conical or more cylindrical PgLPS_1690_ may interact with TLR2 and/or TLR4. However, *P. gingivalis* LPS_1690_ may preferentially bind to TLR4 as five fatty acid chains are sufficient to fully occupy the TLR4 binding pocket as observed previously [40]. In contrast, *P. gingivalis* LPS_1435/1449_,which has strictly cylindrical shape with four fatty-acid chains, might to some extent occupy TLR2 [67]. However, further investigation is required to confirm these points.

*P. gingivalis* possesses multiple mechanisms for the binding and uptake of hemin into the periplasmic and cytoplasmic compartments. Hemin concentration in the vicinity may transduce conformational changes in *P. gingivalis* LPS via regulation of hemin receptors or modification of phosphatases [68]. It has also been shown that *P. gingivalis* grown in high hemin conditions produces predominantly the isoform of LPS with tetra-acylated lipid A structure containing 4-phosphate group, i.e. *P. gingivalis* LPS_1435/1449_. In contrast, under low hemin conditions *P. gingivalis* produces the isoform of LPS with penta-acylated lipid A structure, i.e. *P. gingivalis* LPS_1690_ [56]. Hence, certain micro-environmental conditions like high hemin concentration during inflammation may promote *P. gingivalis* to shift its LPS from the predominant penta-acylated lipid A structure towards more tetra-acylated one. This lipid A transformation has been observed in both laboratory and clinical isolates of *P. gingivalis* [68]. Therefore, it has been suggested that shifting LPS into tetra-acylated lipid A structure may dampen the TLR4-mediated immuno-inflammatory response of gingival tissues, allowing the adaptive pathogen to invade and proliferate in the gingival tissues, thereby leading to progression of periodontal disease. This phenomenon has also been seen in other Gram-negative bacteria such as *Yersinia pestis,* which modifies its lipid A structure from hexa-acylated to a tetra-acylated lipid A during the transition from 27°C to 37°C [69]. This deacylation process bestows the ability of bacterial LPS to dampen the host immune response. Structural modulation of lipid A in other Gram-negative bacteria such as *P. aureginosa* has important clinical implications as well [70].

Within the limitations of the study, the present findings are consistent with other observations [71]–[73], which demonstrates that the tetra- and penta-acylated lipid A structures of *P. gingivalis* LPS interact differentially with TLR2 and TLR4, and critically determine the subsequent activation of the downstream signal transduction cascade that differentially modulates immuno-inflammatory response. This reflects the critical importance of lipid A structural heterogeneity of *P. gingivalis* LPS in activation of TLR receptors and their downstream signal transduction pathways in *P. gingivalis*-host cell interactions. It could be postulated that the ability to alter the lipid A structure of LPS may be a crucial strategy adopted by *P. gingivalis* as a keystone periodontal pathogen to evade innate host defense, thereby contributing to periodontal pathogenesis. The present study sheds new light on what is currently known about the interactions of host cells like HGFs with heterogeneous isoforms of *P. gingivalis* LPS, and contributes to further understanding of the pathogenesis of bacteria-induced inflammatory diseases like periodontal disease, and developing novel preventive and therapeutic approaches to controlling these diseases.

## Experimental Procedures

### Preparation and Purification of *P. gingivalis* LPS

*P. gingivalis* LPS was isolated from *P. gingivalis* ATCC 33277 strain using cold MgCl_2_-ethanol (EtOH) procedure as described previously [28], [43]. LPS purification was undertaken using TRI Reagent approach, as documented previously [74]. Crude LPS was subjected to modified Folch extraction to remove phospholipids and further treated to remove trace amounts of endotoxin proteins preparations detected by enhanced Colloidal gold staining [43]. Lipid A was purified using mild acid hydrolysis as described previously, and the total fatty acid content of LPS was analyzed by Gas chromatography (GC) [75]. Extracted lipid A was then analyzed by negative ion MALDI-TOF MS for the structural determination of lipid A observed [28], [43]. Two detected ion peaks that were clusterd around a mass of 1690 and 1435/1449 designated as penta-acylated *P. gingivalis* LPS_1690_ and tetra acylated *P. gingivalis* LPS_1435/1449_, respectively_._ Highly purified *E. coli* LPS (JM 83 wild type strain) served as positive control.

### HGF Cell Culture

Primary HGFs were purchased from Sciencell research laboratories (Carlsbad, CA, USA) and cultured according to the manufacturer’s instructions. Cells were suspended in fibroblast medium consisting of the basal medium, 2% Fetal Bovine Serum (FBS), fibroblast growth supplement (FGS) and 2% penicillin/streptomycin (P/S), and then incubated with an atmosphere of 5% CO_2_ and 95% air at 37°C [33], [76]. The cultured cells at 3–4 passages, with spindle shaped morphology, were designated as appropriate for the following experiments.

### LPS Stimulation

HGFs were cultured in six-well plates with 1×10^5^ cells per well. While reaching 95% confluence, FM medium was replaced with acf-FM for subsequent dose- and time-dependent experiments. In the dose-dependent assay, cells were stimulated with *P. gingivalis* LPS_1435/1449,_ *P. gingivalis* LPS_1690_ or *E. coli* LPS at various doses (1 ng/ml–10 µg/ml). Based on the results, 1 µg/ml was selected as the appropriate dose for the subsequent time-dependent experiments. In the time-dependent assays, cells were treated with 1 µg/ml of *P. gingivalis* LPS or *E. coli* LPS and incubated for different period of time (2–48 h). Cells without LPS treatment were taken as the controls. Culture supernatants were collected and centrifuged to remove the cell debris and stored in -70°C until further use. The attached cells were then washed with PBS and subjected to RNA and protein extraction, respectively. Total proteins were extracted by using Mammalian protein extraction buffer plus protease and phosphatase inhibitors (Pierce, Thermo Scientific, USA). Cell lysates were collected and centrifuged at 14,000 rpm at 4°C for 15 min to remove the cell debris. The protein concentration was then measured in both cellular proteins and culture supernatants using BCA protein assay kit (Pierce, Thermo scientific, USA).

### Transcriptomic Analysis of TLR Signaling Pathway using PCR-array

In order to explore the holistic view of gene expression in HGFs, upon treatment with *P. gingivalis* LPS_1435/1449_ and LPS_1690_, a panel of 84 genes related to TLR signaling pathway was examined using RT^2^ profiler PCR arrays (PAHS 018C, SA biosciences, Frederick, MD, USA). The complete description of the analyzed genes was listed in Table S2. In order to ensure the high quality of cDNA, reverse transcription reactions were performed using RT^2^ First Strand Kit according to the manufacturer’s protocol (SuperArray, Frederick, MD, USA). Diluted cDNA template was mixed with RT^2^ qPCR Master Mix (SYBR Green/Rox, SA Biosciences) and RNAse-free water (SuperArray Bioscience Corp, Frederick, MD, USA). Then 25 µL of the experimental cocktail were aliquoted to each well of the 96-format PCR array plate containing pre-dispensed gene specific primers. Finally, mRNA was amplified on a StepOne Real-Time PCR system (ABI, Foster City, CA, USA), using the following amplification procedure. After the initial incubation at 95°C for 10 min, 40 cycles of amplification was accomplished with 15 s at 95°C for denaturation and 1 min at 60°C for annealing, respectively. To check the differential expression of related genes, each run was performed in duplicates with reference to the controls. To ensure the reliability, reverse transcription controls (RTC), positive controls (PPC) and genomic DNA controls (GDC) were included in the experiments. The instrument’s software calculated the threshold cycle (Ct) values for all genes tested in the array. Finally, the fold changes in gene expression were calculated for pairwise comparison using the ΔΔCt method from the raw threshold cycle data 2010 [77]. Gene expression was considered up-regulated (fold-changes >1.5) or down-regulated (fold-changes <0.5), and the analysis was carried out using the SA biosciences web-based PCR array data analysis software (SA Biosciences, Frederick, MD, USA).

### Evaluation of Candidate Genes by Real-time qPCR

Real-time qPCR was performed to further examine the candidate genes related to TLR pathway. Total RNA extraction, cDNA synthesis and RT-PCR reaction were performed as mentioned previously [33]. Total RNA was extracted by using RNeasy mini kit (Qiagen, USA) and the RNA concentration was quantified by using the NanoDrop spectrophotometer (Thermo, USA). The extracted RNA was then subjected to cDNA synthesis by using reverse transcriptase-PCR described elsewhere [26]. Q-RT-PCR was performed in StepOne Real-Time PCR System (Applied Biosystems, USA) in at least three separate experiments. Amplification reactions were performed in a final volume of 20 µl containing 10 µl of Power SYBR_Green PCR MasterMIx (Applied Biosystems), 1 µl of cDNA tempelate and 1 µl of each pairs of primers (Sigma). Real-time primer pairs were designed using primer 3 software (NCBI, USA) (Table S3). The amplification efficiencies of the primers used were above 90%. Real-time qPCR reaction conditions were set at 95°C for 10 min followed by 40 cycles at 95°C for 15 s and 60°C for 1 min. The expression level of each gene was normalized to a β-actin (Ct) and fold-changes for each gene were calculated by comparing the LPS-treated test and untreated controls from the Ct values according to the Ct approach [26], [33].

### Detection of TLRs Expression by Confocal Immunofluorescence Microscopy

HGFs were seeded on 12 mm circular cover slips in six-well plates (1×10^6^ cells/well) and cultured overnight in order to achieve over 80% confluent. Afterwards, cells were incubated with 1 µg/ml of either *P. gingivalis* LPS_1435/1449_, *P. gingivalis* LPS_1690_ or *E. coli* LPS for 6 h and 24 h. Cells without any stimulus were taken as controls. After LPS stimulation, cover slips were washed twice in PBS and fixed with 4% (V/V) paraformaldehyde in PBS for 15 min at room temperature. The cover slips were then washed three times in PBS, and permeabilized by treatment with 0.1% Triton X-100 in PBS for 10 min. Following washing three times in PBS, and blocked with PBS containing 3% bovine serum albumin (BSA), plus Tween 20 (0.1% v/v), blocking buffer for 30 min at room temperature, cells were then incubated overnight at 4°C with blocking buffer containing the primary antibodies for TLR4 (polyclonal anti-rabbit TLR4 antibody, 1∶100, Santa Cruz Biotechnology, Santa Cruz, CA, USA) and TLR2 (monoclonal mouse anti-human TLR2 antibody, 1∶100, Abcam). Cells were then washed with 0.1% BSA-PBS and incubated in blocking buffer containing corresponding secondary antibodies (anti-rabbit or anti-mouse IgG conjugated with Alexa fluor 488, 1∶200) for 1 h at room temperature, and excess stain was rinsed off by PBS washes. The cell contour stained for F-actin was detected after 20 min incubation by phalloidin conjugated Alexa fluor 555 (1∶40, Invitrogen, Eugene, Oregone, USA). Next, cells were washed with PBS/TBS and visualized on a confocal laser-scanning microscope (Olympus Fluoview FV 1000; Olympus, Tokyo, Japan) using FV10-ASW 3.0 software for image analysis. For detection of cell nuclei, cells were stained with DAPI (4′6-diamidino-2- phenylindole, dilactate, Invitrogen, USA). Cells treated with IgG isotype control (R & D systems) instead of the primary antibody served as the negative control.

### Pathway-focused Western Blot Analysis

Western blot analysis was performed to examine the expression of TLR2, TLR4 and other key molecules related to major signal transduction pathways such as p-IκBα, p-p65, p-p38 MAPK, p-ERK, p-JNK and p-AKT. HGFs were serum starved for 24 h and then stimulated with 1 µg/ml of *P. gingivalis* LPS_1435/1449_, *P. gingivalis* LPS_1690_ or *E. coli* LPS, for 5, 15, 30, 60 and 120 min. Western blots were performed according to the standard protocol, which were used in previous studies [78]. All the pathway molecules were examined using repeated stripping technique for each blot, in order to minimize the batch-to-batch variation. Initially, each blot was probed for phosphorylated proteins, followed by stripping and re-probing with the appropriate probe for total proteins. α-Tubulin was used as the internal loading control. In brief, 40 µg of protein lysates were separated by 10% SDS-PAGE and transferred onto PVDF (Polyvinylidene difluoride) membranes (Roche, USA) by using the Mini-PROTEAN Tetra electrophoresis system and the Mini Trans-Blot transfer system (Bio-Rad, USA). Following the transfer, blots were blocked with protein-free T20 (TBS) blocking buffer (Thermo scientific, USA) at room temperature for 1 h and incubated with primary antibody at 4°C while shaking overnight. Primary antibodies were all obtained against monoclonal rabbit anti-human antibodies; TLR4 antibody (1∶1000, Santa Cruz), TLR2 antibody (1∶1000, Cell Signaling), phospho IκBα (pIκBα)(1∶1000, Cell Signaling), IκBα antibody (1∶1000, Cell Signaling), phospho NF-κB p65 (1∶1000, Cell Signaling), NF-κB p65 (1∶1000, Cell Signaling), phospho-p38 antibody (pP38MAPK) (1∶1000, Cell Signaling), p38MAPK antibody (1∶1000, Cell Signaling), phospho-SAPK/JNK p-JNK antibody (1∶1000, Cell Signaling), SAPK/JNK antibody (1∶1000, Cell Signaling), phospho-ERK1/2 antibody (1∶2000, Cell Signaling), ERK1/2 antibody (1∶1000, Cell Signaling), phospho-AKT antibody (1∶1000, Cell Signaling), and AKT antibody (1∶1000, Cell Signaling). α-Tubulin (1∶2000, Cell Signaling) was used as the internal loading control. After being washed with the washing buffer, the blots were incubated with horseardish peroxidase (HRP) conjugated goat-anti-rabbit IgG (1∶10000, Cell Signaling) at room temperature for 1 h, then the bound immune-complexes were detected using ECL reagent (super signal west pico chemiluminescent kit, Thermo Scientific, USA). Detected bands were scanned on a calibrated densitometer (GS-800, Bio-Rad, Hercules, CA, USA) and the integrated density of each band was quantified using Image J software-based analysis (http://rsb.info.nih.gov/ij/).

### Analysis of NF-κB Nuclear Translocation

Activation and translocation of NF-κB were observed by confocal immunofluorescence assay as mentioned previously. After LPS stimulation for 15 and 60 min, cells were fixed, blocked and incubated with rabbit anti-phospho NF-κB p65 (1∶100, Cell Signaling) over night at 4°C. After three washes in 0.1% BSA-PBS, the cells were incubated with FITC conjugated goat anti-rabbit secondary antibody at room temperature for 60 min. After rinsing in PBS, cells were counterstained with DAPI (10 µg/ml) for 5 min. The slides were then washed, air-dried and mounted with fluorescent mounting medium and visualized on a confocal laser-scanning microscope (Olympus Fluoview FV 1000; Olympus, Tokyo, Japan) using FV10-ASW 3.0 software for image analysis. Negative controls were established by omitting primary antibody.

### Blocking Assays of TLR2 and TLR4

Neutralization of TLRs was achieved by using TLR-specific blocking antibodies. HGFs were grown in six-well tissue culture plates until 90% confluent as described above. Then the cells were incubated for 1 h with serum free fresh media containing 20 µg/ml of anti-human TLR2 antibody (eBioscience, San Diego, USA) and 20 µg/ml of anti-human TLR4 antibody (eBioscience) using 20 µg/ml of mouse IgG2_a_ isotype control (Biolegend, San Diego, CA, USA) as the negative control, prior to the addition of LPS. Afterwards, cells were challenged with 1 µg/ml of either *P. gingivalis* LPS_1435/1449_, *P. gingivalis* LPS_1690_ or *E. coli* LPS for 12 h. Cells incubated with medium alone was considered as the negative control, while cells incubated with LPS without prior incubation with TLR antibody were used as the positive control. After stimulation, culture media supernatants were collected for cytokine assays and the cells were harvested for extraction of total mRNA.

### Blocking Assays of Signal Transduction Pathways

The functional roles of NF-κB, p38 MAPK and ERK involved in the interactions of HGFs with *P. gingivalis* LPS_1690_ or *E. coli* LPS were examined using pathway-specific kinases inhibitors. To block the specific kinase activity, cells were pretreated with following specific kinase inhibitors for 1 h before stimulation with LPS: 10 µmol/L of the IKK-β inhibitor, IKK-2 inhibitor IV (Merck, USA), 10 µmol/L of the p38 MAPK inhibitor, SB202190 (Calbiochem Biosciences Inc, La Jolla, CA, USA), and 15 µmol/L of the ERK (MEK1) inhibitor, U1026 (Cell Signaling). Each inhibitor was dissolved in DMSO and diluted in DPBS. Afterwards, LPS was added to the medium and cells were incubated for another 12 h. Culture media supernatants and RNA were used for ELISA and real-time qPCR analysis, respectively. Cells incubated only with LPS, without adding any kinase inhibitors, were regarded as positive controls, whereas those treated with culture medium alone served as the negative controls. To examine the effects of these inhibitors on the basal expression of cytokines, cells were treated with kinase inhibitors alone.

### Assay of IL-6 and IL-8 by ELISA

The expression profiles of IL-6 and IL-8 were analyzed in culture supernatants using specific human ELISA kits (DuoSet, R&D Systems, Minneapolis, MN, USA) in triplicates following the manufacturer’s instructions. The minimal detectable concentrations of IL-6 and IL-8 were 0.70 pg/ml and 3.5 pg/ml, respectively. No cross-reactivity or interference was observed with recombinant IL-6 and IL-8. The absorbance values for the ELISA assays were determined by a microplate reader (Victor, Vienna, VA, USA) at an optical absorbance of 450 nm. The final concentration was determined with reference to a standard curve.

### Statistical Analysis

All experiments were repeated in at least three assays for real-time qPCR, western blot and two assays for ELISA. All values were presented as the mean ±SD. The statistical significance of difference between the data sets from the dose-dependent assay was evaluated by student *t*-test, one-way analysis of variance (ANOVA) and post-hoc testing with Bonferroni and LSD methods, as appropriate. Additionally, repeated measures ANOVA were used to determine the differences between data sets from the time-dependent assay. A *p-*value <0.05 was considered statistically significant. All statistical analysis was performed using a software program (SPSS 19.0, SPSS Inc, Chicago, IL, USA).

## Supporting Information

Figure S1 **Confocal images of TLR2 (S1.1) and TLR4 (S1.2) expression in HGFs following LPS stimulation for 24 h.** HGFs were left untreated (A) or stimulated with 1 µg/ml of *P. gingivalis* (Pg) LPS_1435/1449_ (PgLPS_1435/1449_) (B) PgLPS_1690_ (C) and *E. coli* LPS (D). Negative control: E. Cells were then permeabilized with 0.1% Triton X-100 and subsequently stained with primary antibodies against TLR2, TLR4 and the correspondent secondary antibody labeled Alexa fluor 488 anti-rabbit, and subsequently stained with alexa fluor 555 phalloidin for F-actin. Merged images present the combined TLR2 or TLR4, F-actin, and nuclear staining (DAPI). One representative experiment from three independent experiments is shown. Bar = 50 µm or 100 µm.
(TIF)

Figure S2 ***P. gingivalis*** **(Pg) LPS -induced gene expression of inflammatory mediators in HGFs.** The cells were treated with PgLPS at 1 µg/mL or culture medium alone for 24 h. Total RNA was extracted and reverse transcribed into cDNA templates. The templates used in PCR array were pooled equally from triplicate samples. Representative heat maps showing the fold-changes of each gene in PgLPS_1435_ (A)- and PgLPS_1690_ (B)-treated HGFs with reference to the controls. Genes that were upregulated over 2 folds are shown in red color and those down regulated by 0.5 folds are shown in green color.
(TIF)

Figure S3 ***P. gingivalis*** **(Pg) LPS_1690_ induced the mRNA expression of inflammatory mediators in HGFs.** The cells were stimulated with PgLPS and *E. coli* LPS (1 µg/mL) for 24 h. The harvested RNA was subjected to real-time quantitative PCR analysis. Fold increase of genes were analyzed relative to the internal control β-Actin, including GM-CSF (A), CXCL10 (B), IL-6 (C) and IL-8 (D). Each bar represents the mean±SD of three independent experiments with three replicates. *Significant difference with a *p*-value <0.05 as compared with the controls without LPS treatment.
(TIF)

Figure S4 **Kinetics of protein kinase B (PKB) or AKT phosphorylation in HGFs.** The cells were stimulated with *P. gingivalis* (Pg) LPS_1435/1449_ **(**PgLPS_1435/1449_) (A), PgLPS_1690_ (B) and *E. coli* LPS (C) at 1 µg/mL for the indicated periods of time. Cell extracts were prepared and the sample aliquots containing 40 µg of protein were separated by SDS-polyacrylamide gel electrophoresis and immunoblotted with anti-phopho AKT specific antibodies. Fold increase values of p-AKT optical density (arbitrary units over control after normalization to the loading control (total AKT) are shown in the graphs (D). The data shown here are from a representative experiment repeated three times with similar results**.** *Significant difference with a *p*-value <0.05 as compared with the controls without LPS treatment.
(TIF)

Table S1 Differential expression profile of genes associated with TLR signal transduction in HGF. The cells were treated with *P. gingivalis* (Pg) LPS_1435/1449_ (PgLPS_1435/1449_) and PgLPS_1690_ (1 µg/mL) for 24 h. After the stimulation, mRNA was extracted from cellular fraction and reverse transcribed to cDNA. Pathway-focused PCR gene array was adopted to analyse the cDNA corresponding to 84 inflammation-associated genes quantified with qRT-PCR. Relative expression was analysed comparing the LPS treated cells with the cDNA prepared from the controls. The fold-changes in gene expression in the *P. gingivalis* LPS-treated cells versus control cells are listed. Genes that were upregulated over 2 folds are marked in red color and those down regulated by 0.5 folds are highlighted in blue color.
(DOCX)

Table S2 Genes included in the TLR signaling pathway RT-PCR array kit (SA Biosciences). A total of 84 genes related to TLR signaling family were analyzed, including adaptor and effector proteins, members of the NF-κB, JNK/p38, IRF and JAK/STAT signaling pathways as well as downstream pathway genes.
(DOCX)

Table S3 Nucleotide sequence of primers for real-time PCR. Quantitative real time (QRT) PCR was performed using custom-designed primers for the cell surface receptors, adaptor molecules and pro-inflammatory cytokines using purified RNA from HGFs stimulated with *P. gingivalis* LPS and *E. coli* LPS.
(DOCX)
