# Supplementary figures and images for: Tetra- and Penta-Acylated Lipid A Structures of Porphyromonas gingivalis LPS Differentially Activate TLR4-Mediated NF-κB Signal Transduction Cascade and Immuno-Inflammatory Response in Human Gingival Fibroblasts
Source: PLoS One. 2013 Mar 12;8(3):e58496. doi: 10.1371/journal.pone.0058496 (PMC3595299; doi:10.1371/journal.pone.0058496)

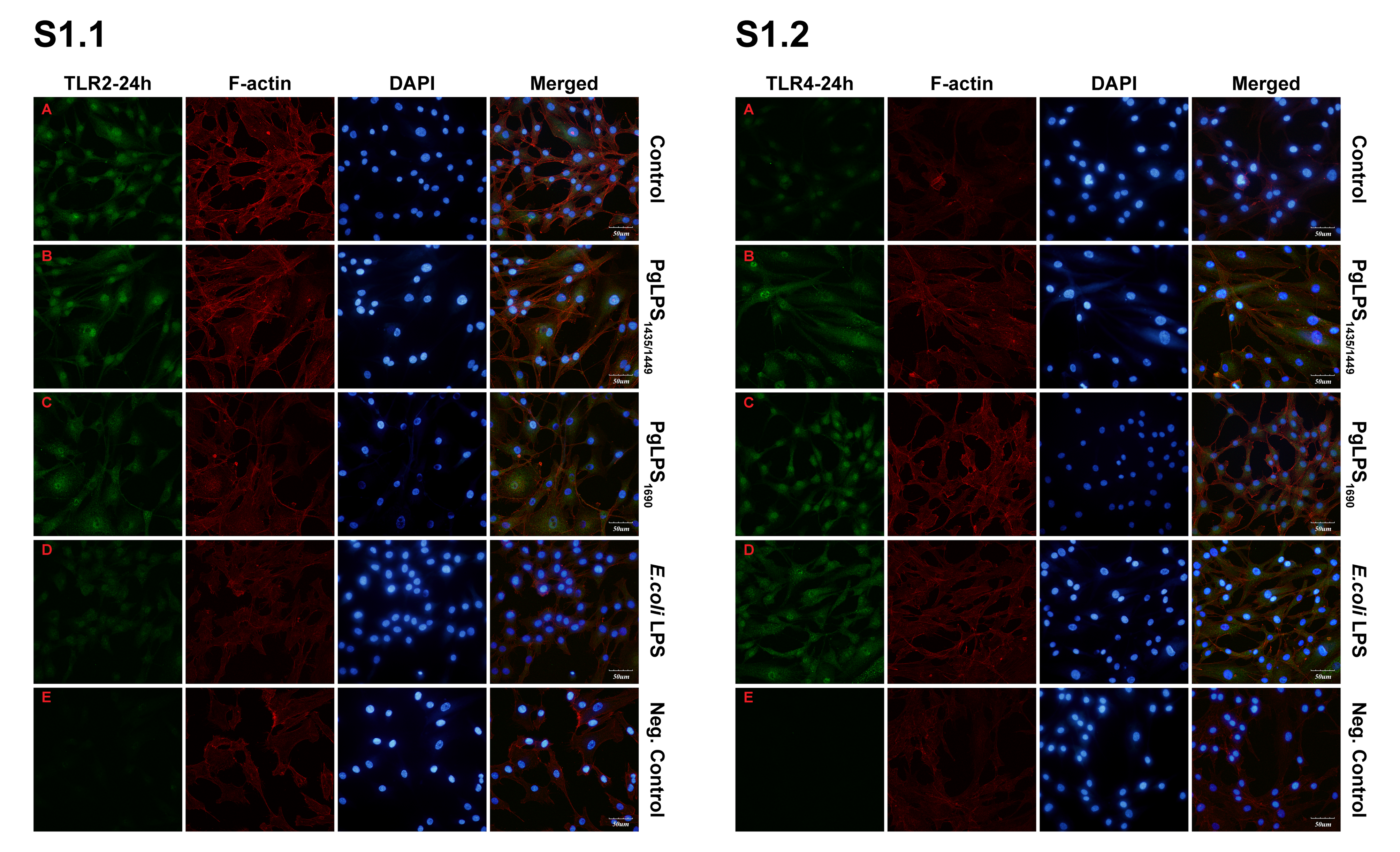

Supplement: Figure S1 — Confocal images of TLR2 (S1.1) and TLR4 (S1.2) expression in HGFs following LPS stimulation for 24 h. HGFs were left untreated (A) or stimulated with 1 µg/ml of P. gingivalis (Pg) LPS1435/1449 (PgLPS1435/1449) (B) PgLPS1690 (C) and E. coli LPS (D). Negative control: E. Cells were then permeabilized with 0.1% Triton X-100 and subsequently stained with primary antibodies against TLR2, TLR4 and the correspondent secondary antibody labeled Alexa fluor 488 anti-rabbit, and subsequently stained with alexa fluor 555 phalloidin for F-actin. Merged images present the combined TLR2 or TLR4, F-actin, and nuclear staining (DAPI). One representative experiment from three independent experiments is shown. Bar = 50 µm or 100 µm. (TIF) [file pone.0058496.s001.tif]

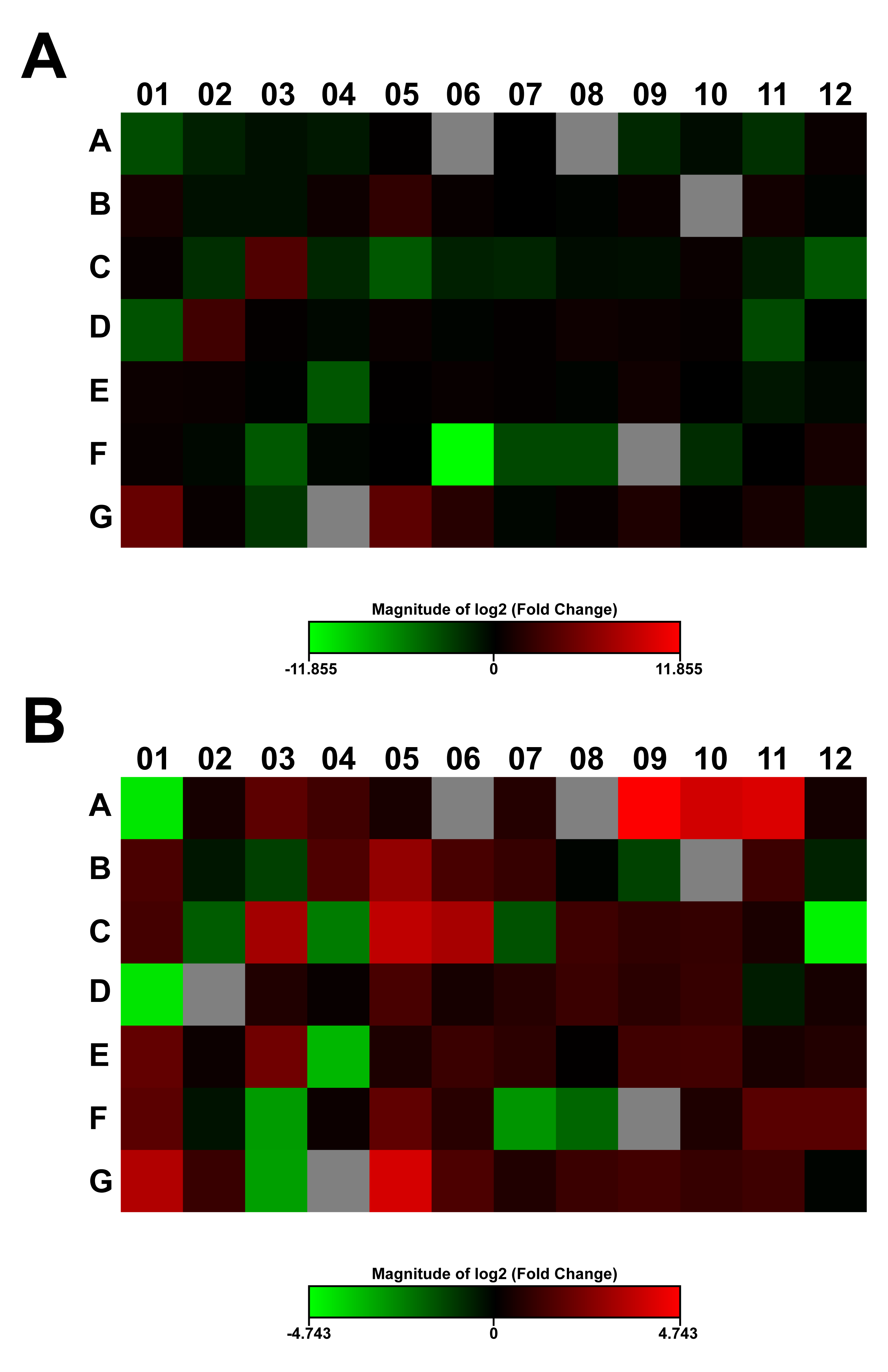

Supplement: Figure S2 — P. gingivalis (Pg) LPS -induced gene expression of inflammatory mediators in HGFs. The cells were treated with PgLPS at 1 µg/mL or culture medium alone for 24 h. Total RNA was extracted and reverse transcribed into cDNA templates. The templates used in PCR array were pooled equally from triplicate samples. Representative heat maps showing the fold-changes of each gene in PgLPS1435 (A)- and PgLPS1690 (B)-treated HGFs with reference to the controls. Genes that were upregulated over 2 folds are shown in red color and those down regulated by 0.5 folds are shown in green color. (TIF) [file pone.0058496.s002.tif]

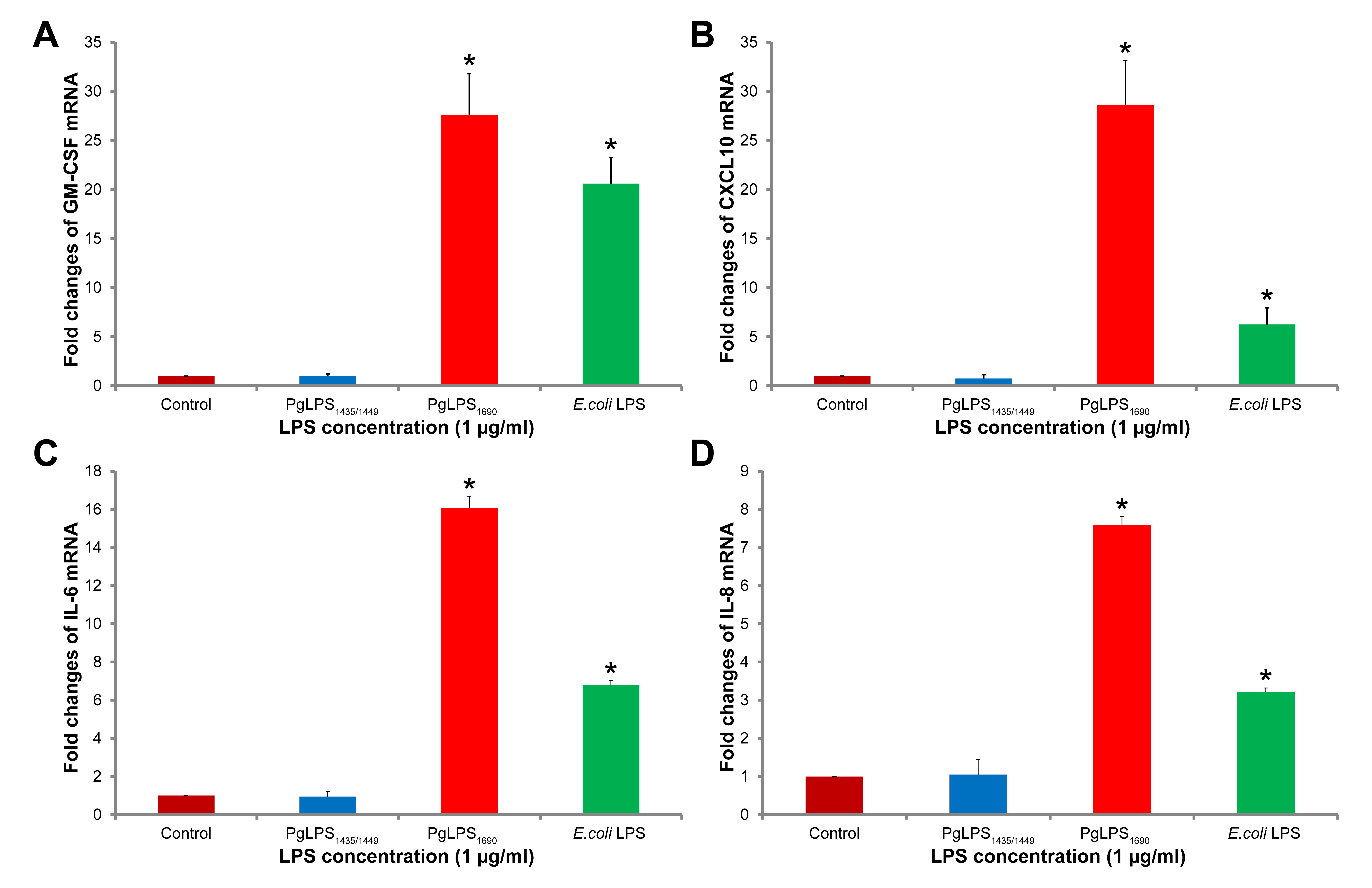

Supplement: Figure S3 — P. gingivalis (Pg) LPS1690 induced the mRNA expression of inflammatory mediators in HGFs. The cells were stimulated with PgLPS and E. coli LPS (1 µg/mL) for 24 h. The harvested RNA was subjected to real-time quantitative PCR analysis. Fold increase of genes were analyzed relative to the internal control β-Actin, including GM-CSF (A), CXCL10 (B), IL-6 (C) and IL-8 (D). Each bar represents the mean±SD of three independent experiments with three replicates. *Significant difference with a p-value <0.05 as compared with the controls without LPS treatment. (TIF) [file pone.0058496.s003.tif]

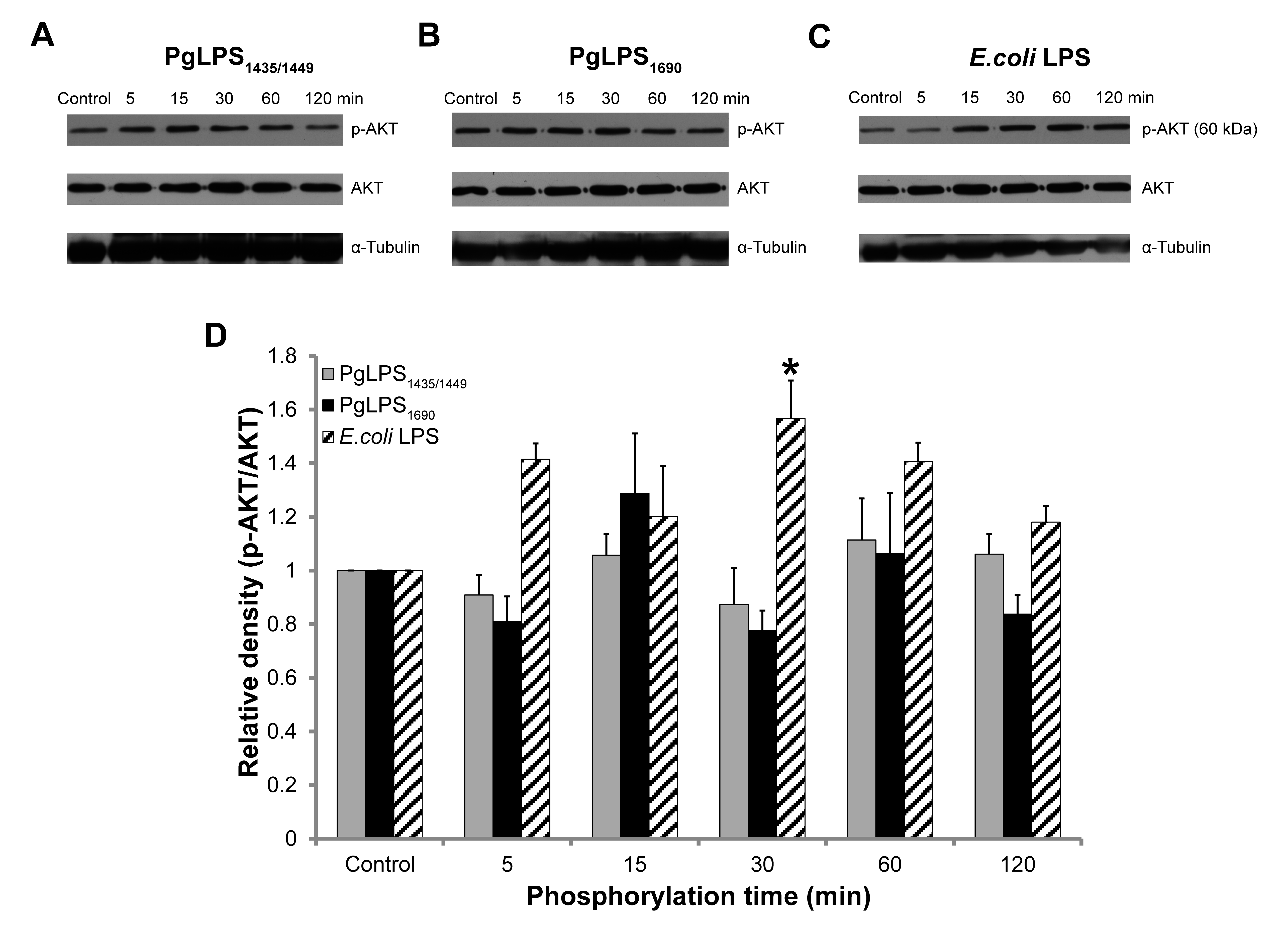

Supplement: Figure S4 — Kinetics of protein kinase B (PKB) or AKT phosphorylation in HGFs. The cells were stimulated with P. gingivalis (Pg) LPS1435/1449 (PgLPS1435/1449) (A), PgLPS1690 (B) and E. coli LPS (C) at 1 µg/mL for the indicated periods of time. Cell extracts were prepared and the sample aliquots containing 40 µg of protein were separated by SDS-polyacrylamide gel electrophoresis and immunoblotted with anti-phopho AKT specific antibodies. Fold increase values of p-AKT optical density (arbitrary units over control after normalization to the loading control (total AKT) are shown in the graphs (D). The data shown here are from a representative experiment repeated three times with similar results. *Significant difference with a p-value <0.05 as compared with the controls without LPS treatment. (TIF) [file pone.0058496.s004.tif]
