# Supplementary material for: Tetra- and Penta-Acylated Lipid A Structures of Porphyromonas gingivalis LPS Differentially Activate TLR4-Mediated NF-κB Signal Transduction Cascade and Immuno-Inflammatory Response in Human Gingival Fibroblasts
Source: PLoS One. 2013 Mar 12;8(3):e58496. doi: 10.1371/journal.pone.0058496 (PMC3595299; doi:10.1371/journal.pone.0058496)
Supplement: Table S1 — Differential expression profile of genes associated with TLR signal transduction in HGF. The cells were treated with P. gingivalis (Pg) LPS1435/1449 (PgLPS1435/1449) and PgLPS1690 (1 µg/mL) for 24 h. After the stimulation, mRNA was extracted from cellular fraction and reverse transcribed to cDNA. Pathway-focused PCR gene array was adopted to analyse the cDNA corresponding to 84 inflammation-associated genes quantified with qRT-PCR. Relative expression was analysed comparing the LPS treated cells with the cDNA prepared from the controls. The fold-changes in gene expression in the P. gingivalis LPS-treated cells versus control cells are listed. Genes that were upregulated over 2 folds are marked in red color and those down regulated by 0.5 folds are highlighted in blue color. (DOCX) [file pone.0058496.s005.docx]

**Table S1. Differential expression profile of genes associated with TLR signal transduction in HGFs stimulated by *P. gingivalis* (Pg) LPS_1435/1449_ (PgLPS_1435/1449_) and PgLPS_1690_.** The genes upregulated (fold changes ≥ 2.0) are highlighted in bold and those downregulated (fold changes ≤ 0.5) are marked in italics.

| **Position** | **Gene symbols** | **Gene names** | **Fold difference (LPS/control)** | |
| --- | --- | --- | --- | --- |
|  |  |  | **Pg_1435/1449_** | **Pg_1690_** |
| A01 | BTK | Bruton agammaglobulinemia tyrosine kinase | *0.08* | *0.05* |
| A02 | CASP8 | Caspase 8, apoptosis-related cysteine peptidase | *0.35* | 1.34 |
| A03 | CCL2 | Chemokine (C-C motif) ligand 2 | 0.58 | **3.25** |
| A04 | CD14 | CD14 molecule | *0.45* | **2.26** |
| A05 | CD86 | CD86 molecule | 0.77 | 0.54 |
| A06 | CHUK | Conserved helix-loop-helix ubiquitous kinase | 1.03 | 1.59 |
| A07 | CLEC4E | C-type lectin domain family 4, member E | 0.77 | 0.54 |
| A08 | CSF2 | Colony stimulating factor 2 (granulocyte-macrophage) | *0.28* | **26.77** |
| A09 | CSF3 | Colony stimulating factor 3 (granulocyte) | 0.67 | **14.91** |
| A10 | CXCL10 | Chemokine (C-X-C motif) ligand 10 | *0.21* | **17.27** |
| A11 | ELK1 | ELK1, member of ETS oncogene family | **2.05** | **2.62** |
| A12 | FADD | Fas (TNFRSF6)-associated via death domain | 0.61 | 0.76 |
| B01 | FOS | V-fos FBJ murine osteosarcoma viral oncogene homolog | 0.6 | *0.43* |
| B02 | HMGB1 | High-mobility group box 1 | 1.6 | **2.76** |
| B03 | HRAS | V-Ha-ras Harvey rat sarcoma viral oncogene homolog | **4.66** | **6.74** |
| B04 | HSPA1A | Heat shock 70kDa protein 1A | 1.28 | **2.56** |
| B05 | HSPD1 | Heat shock 60kDa protein 1 (chaperonin) | 0.98 | 1.99 |
| B06 | IFNA1 | Interferon, alpha 1 | 0.89 | 0.94 |
| B07 | IFNB1 | Interferon, beta 1, fibroblast | 1.39 | *0.42* |
| B08 | IFNG | Interferon, gamma | 0.77 | 0.54 |
| B09 | IKBKB | Inhibitor of kappa light polypeptide gene enhancer in B-cells, kinase beta | 1.73 | **2.18** |
| B10 | IL10 | Interleukin 10 | 0.9 | 0.65 |
| B11 | IL12A | Interleukin 12A | 1.33 | **2.38** |
| B12 | IL1B | Interleukin 1, beta | **13.47** | **8.32** |
| C01 | IL2 | Interleukin 2 | *0.29* | *0.2* |
| C02 | IL6 | Interleukin 6 (interferon, beta 2) | *0.06* | **11.93** |
| C03 | IL8 | Interleukin 8 | *0.35* | **8.64** |
| C04 | IRAK1 | Interleukin-1 receptor-associated kinase 1 | *0.32* | *0.34* |
| C05 | IRAK2 | Interleukin-1 receptor-associated kinase 2 | 0.68 | **2.25** |
| C06 | IRF1 | Interferon regulatory factor 1 | 0.62 | 1.86 |
| C07 | IRF3 | Interferon regulatory factor 3 | 1.39 | 1.95 |
| C08 | JUN | Jun oncogene | *0.41* | 1.39 |
| C09 | LTA | Lymphotoxin alpha (TNF superfamily, member 1) | *0.06* | *0.04* |
| C10 | CD180 | CD180 molecule | *0.07* | *0.05* |
| C11 | LY86 | Lymphocyte antigen 86 | **8.07** | 0.54 |
| C12 | LY96 | Lymphocyte antigen 96 | 1.11 | 1.49 |
| D01 | MAP2K3 | Mitogen-activated protein kinase kinase 3 | 0.77 | 1.12 |
| D02 | MAP2K4 | Mitogen-activated protein kinase kinase 4 | 1.38 | **2.55** |
| D03 | MAP3K1 | Mitogen-activated protein kinase kinase kinase 1 | 0.9 | 1.31 |
| D04 | MAP3K7 | Mitogen-activated protein kinase kinase kinase 7 | 1.12 | 1.61 |
| D05 | MAP3K7IP | Mitogen-activated protein kinase kinase kinase 7 interacting protein 1 | 1.61 | **2.10** |
| D06 | MAP4K4 | Mitogen-activated protein kinase kinase kinase kinase 4 | 1.37 | 1.71 |
| D07 | MAPK8 | Mitogen-activated protein kinase 8 | 1.25 | 1.99 |
| D08 | MAPK8IP3 | Mitogen-activated protein kinase 8 interacting protein 3 | *0.09* | 0.7 |
| D09 | MYD88 | Myeloid differentiation primary response gene (88) | 0.98 | 1.32 |
| D10 | NFKB1 | Nuclear factor of kappa light polypeptide gene enhancer in B-cells 1 | 1.48 | **3.55** |
| D11 | NFKB2 | Nuclear factor of kappa light polypeptide gene enhancer in B-cells 2 (p49/p100) | 1.39 | 1.16 |
| D12 | NFKBIA | Nuclear factor of kappa light polypeptide gene enhancer in B-cells inhibitor, alpha | 0.95 | **4.25** |
| E01 | NFKBIL1 | Nuclear factor of kappa light polypeptide gene enhancer in B-cells inhibitor-like 1 | *0.06* | *0.09* |
| E02 | NFRKB | Nuclear factor related to kappaB binding protein | 1.08 | 1.42 |
| E03 | NR2C2 | Nuclear receptor subfamily 2, group C, member 2 | 1.28 | **2.10** |
| E04 | PELI1 | Pellino homolog 1 (Drosophila) | 1.15 | 1.75 |
| E05 | PPARA | Peroxisome proliferator-activated receptor alpha | 0.88 | 1.03 |
| E06 | PRKRA | Protein kinase, interferon-inducible double stranded RNA dependent activator | 1.66 | **2.28** |
| E07 | PTGS2 | Prostaglandin-endoperoxide synthase 2 | 0.98 | **2.32** |
| E08 | REL | V-rel reticuloendotheliosis viral oncogene homolog | *0.49* | 1.37 |
| E09 | RELA | V-rel reticuloendotheliosis viral oncogene homolog A | 0.77 | 1.57 |
| E10 | RIPK2 | Receptor-interacting serine-threonine kinase 2 | 1.33 | **3.20** |
| E11 | SARM1 | Sterile alpha and TIR motif containing 1 | 0.76 | 0.79 |
| E12 | SIGIRR | Single immunoglobulin and toll-interleukin 1 receptor (TIR) domain | *0.06* | *0.13* |
| F01 | ECSIT | ECSIT homolog (Drosophila) | 0.84 | 1.18 |
| F02 | TBK1 | TANK-binding kinase 1 | 1.01 | **3.42** |
| F03 | TICAM2 | Toll-like receptor adaptor molecule 2 | *0.1* | 1.66 |
| F04 | TIRAP | Toll-interleukin 1 receptor (TIR) domain containing adaptor protein | *0.1* | *0.15* |
| F05 | TLR1 | Toll-like receptor 1 | *0.1* | *0.27* |
| F06 | TLR10 | Toll-like receptor 10 | 0.77 | 0.54 |
| F07 | TLR2 | Toll-like receptor 2 | *0.24* | 1.48 |
| F08 | TLR3 | Toll-like receptor 3 | 1.02 | **3.09** |
| F09 | TLR4 | Toll-like receptor 4 | **2.04** | **3.14** |
| F10 | TLR5 | Toll-like receptor 5 | **26.89** | **10.02** |
| F11 | TLR6 | Toll-like receptor 6 | 1.27 | **2.07** |
| F12 | TLR7 | Toll-like receptor 7 | *0.18* | *0.13* |
| G01 | TLR8 | Toll-like receptor 8 | 0.77 | 0.54 |
| G02 | TLR9 | Toll-like receptor 9 | **19.96** | **15.58** |
| G03 | TNF | Tumor necrosis factor (TNF superfamily, member 2) | **3.49** | **2.65** |
| G04 | TNFRSF1A | Tumor necrosis factor receptor superfamily, member 1A | 0.83 | 1.51 |
| G05 | TOLLIP | Toll interacting protein | 1.33 | **2.12** |
| G06 | TRAF6 | TNF receptor-associated factor 6 | **2.57** | **2.35** |
| G07 | TICAM1 | Toll-like receptor adaptor molecule 1 | 1.08 | 1.99 |
| G08 | UBE2N | Ubiquitin-conjugating enzyme E2N (UBC13 homolog) | **2.05** | **2.20** |
| G09 | UBE2V1 | Ubiquitin-conjugating enzyme E2 variant 1 | 0.52 | 0.94 |
