# Supplementary material for: Tetra- and Penta-Acylated Lipid A Structures of Porphyromonas gingivalis LPS Differentially Activate TLR4-Mediated NF-κB Signal Transduction Cascade and Immuno-Inflammatory Response in Human Gingival Fibroblasts
Source: PLoS One. 2013 Mar 12;8(3):e58496. doi: 10.1371/journal.pone.0058496 (PMC3595299; doi:10.1371/journal.pone.0058496)
Supplement: Table S2 — Genes included in the TLR signaling pathway RT-PCR array kit (SA Biosciences). A total of 84 genes related to TLR signaling family were analyzed, including adaptor and effector proteins, members of the NF-κB, JNK/p38, IRF and JAK/STAT signaling pathways as well as downstream pathway genes. (DOCX) [file pone.0058496.s006.docx]

**Table S2. Genes included in the TLR signaling pathway RT-PCR array kit (SA Biosciences, USA).**

| **Description** | **Gene names** |
| --- | --- |
| **Toll-Like Receptors** | CD180 (LY64), SIGIRR, TLR1, TLR2, TLR3, TLR4, TLR5, TLR6, TLR7, TLR8, TLR9, TLR10 |
| **Pathogen-Specific Responses:**  Bacterial | CCL2 (MCP-1), CD14, CD180 (LY64), FOS, HRAS, IL10, IL12A, IL1B, IL6, IL8, IRAK1, HMGB1, HSPA1A (HSP70 1A), JUN, LTA (TNFB), LY86 (MD-1), LY96, NFKBIA (IKBA/MAD3), PTGS2 (COX2), RELA, RIPK2, TLR2, TLR4, TLR6, TNFRSF1A, TICAM1 (TRIF). |
| Viral | EIF2AK2 (PRKR), IFNB1, IFNG, IL12A, IL6, IRF3, PRKRA, RELA, TBK1, TLR3, TLR7, TLR8, TNF, TICAM1 (TRIF). |
| Fungal/Parasitic | CLEC4E, HRAS, HSPA1A (HSP70 1A), IL8, TLR2, TIRAP. |
|  |  |
| **TLR Signaling** Negative Regulation | SARM1, SIGIRR, TOLLIP |
| TICAM1 (TRIF)-Dependent (MYD88-Independent): | IRF3, MAP3K7 (TAK1), TAB1, NR2C2, PELI1, TBK1, TICAM2, TLR3, TLR4, TRAF6, TICAM1 (TRIF). |
| MYD88-Dependent | IRAK1, IRAK2, MAP3K7 (TAK1), TAB1, MYD88, NR2C2, TIRAP, TLR1, TLR10, TLR2, TLR4, TLR5, TLR6, TLR7, TLR8, TLR9, TRAF6. |
| **Downstream Pathways and Target Genes:** NFκB Pathway | BTK, CASP8, CHUK (IKKa), ECSIT (SITPEC), FADD, IKBKB, IL10, IL1B, IRAK1, IRAK2, IRF3, LY96, MAP3K1 (MEKK), MAP3K7, MAP4K4, NFKB1, NFKB2, NFKBIA (IKBA/MAD3), NFKBIL1, NFRKB, PPARA, REL, RELA, TNF, TNFRSF1A, UBE2N, UBE2V1. |
| JNK/p38 Pathway | ELK1, FOS, IL1B, JUN, MAP2K3 (MEK3), MAP2K4 (JNKK1), MAP3K1 (MEKK), MAP3K7, MAPK8 (JNK1), MAPK8IP3, TNF. |
| JAK/STAT Pathway | CCL2 (MCP-1), CSF2 (GM-CSF), IFNG, IL12A, IL2, IL6. |
| Interferon Regulatory Factor (IRF) Pathway | CXCL10 (INP10), IFNA1, IFNB1, IFNG, IRF1, IRF3, TBK1 |
| Cytokine-Mediated Signaling Pathway | CCL2 (MCP-1), CSF3 (GCSF), IL1A, IL1B, IL6, IRAK1, IRAK2, RELA, SIGIRR, TNF, TNFRSF1A. |
|  |  |
| **Regulation of Adaptive Immunity** | CD80, CD86, HSPD1, IFNG, IL10, IL12A, IL1B, IL2, MAP3K7, TRAF6 |
| **Adaptors & TLR Interacting Proteins** | BTK, CD14, HMGB1, HRAS, HSPA1A (HSP70 1A), HSPD1, LY86 (MD-1), LY96 (MD-2), MAPK8IP3, MYD88, PELI1, RIPK2, SARM1, TICAM1 (TRIF), TICAM2 (TRAM), TIRAP, TOLLIP |
| **Effectors** | CASP8 (FLICE), EIF2AK2 (PRKR), FADD, IRAK1, IRAK2, MAP3K7 (TAK1), TAB1, NR2C2, PPARA, PRKRA, ECSIT (SITPEC), TRAF6, UBE2N, UBE2V1. |
