# Supplementary material for: Tetra- and Penta-Acylated Lipid A Structures of Porphyromonas gingivalis LPS Differentially Activate TLR4-Mediated NF-κB Signal Transduction Cascade and Immuno-Inflammatory Response in Human Gingival Fibroblasts
Source: PLoS One. 2013 Mar 12;8(3):e58496. doi: 10.1371/journal.pone.0058496 (PMC3595299; doi:10.1371/journal.pone.0058496)
Supplement: Table S3 — Nucleotide sequence of primers for real-time PCR. Quantitative real time (QRT) PCR was performed using custom-designed primers for the cell surface receptors, adaptor molecules and pro-inflammatory cytokines using purified RNA from HGFs stimulated with P. gingivalis LPS and E. coli LPS. (DOCX) [file pone.0058496.s007.docx]

**Table S3. Nucleotide sequence of real-time PCR primers.**

| **Primers** | **Sequence (5'-3')** |
| --- | --- |
| TLR2- F | CTT TCA CTG CTT TCA ACT GGT A |
| TLR2-R | TTG CGG TCA CAA GAC AGA G |
| TLR4-F | GAC TTG CGG GTT CTA CAT CA |
| TLR4-R | GAG GTG GCT TAG GCT CTG ATA |
| CD14-F | TGC CCT GCA GAA TCC TTC CTG T |
| CD14-R | TCG GCT GCC TCT GAC AGT TTA T |
| LBP-F | CTG AAG CCA GGA AAG GTA AAA G |
| LBP-R | GCA GCC CAA GGT CGT AGA |
| MD2-F | TAT TGG GTC TGC AAC TCA T |
| MD2-R | CTC CCA GAA ATA GCT TCA A |
| MYD88-F | GAG CAC AGA TTC CTC CTA CAA C |
| MYD88-F | GCG ACT ACA CCA ACC CCT |
| GM- CSF-F | GCA TTC CTC CTG ATC CCA GA |
| GM-CSF -R | CCT GGA GTC AAA CCT CAC ATT |
| CXCL10-F | CTG AAT CCA GAA TCG AAG GCC |
| CXCL10-R | TGA TCG ATT TTG CTC CCC |
| IL6-F | AAT CAT CAC TGG TCT TTT GGA G |
| IL6-R | GCA TTT GTG GTT GGG TCA |
| IL8-F | GAA CCA TCT CAC TGT GTG TAA A |
| IL8-R | CAC TCC TTG GCA AAA CTG |
| ActinB -F | TTG GCA ATG AGC GGT T |
| ActinB -R | AGT TGA AGG TAG TTT CGT GGA T |
